# The Analysis Portal and the Swedish LifeWatch e-infrastructure for biodiversity research

**DOI:** 10.3897/BDJ.4.e7644

**Published:** 2016-03-23

**Authors:** Sonja Leidenberger, Martin Käck, Björn Karlsson, Oskar Kindvall

**Affiliations:** ‡ ArtDatabanken, Swedish Species Information Centre, SLU, Uppsala, Sweden

**Keywords:** Analysis Portal, biodiversity informatics, e-infrastructure, e-sciences, Swedish LifeWatch, web services

## Abstract

**Background:**

During the last years, more and more online portals were generated and are now available for ecologists to run advanced models with extensive data sets. Some examples are the Biodiversity Virtual e-Laboratory (BioVel) Portal (https://portal.biovel.eu) for ecological niche modelling and the Mobyle SNAP Workbench (https://snap.hpc.ncsu.edu) for evolutionary and population genetics analysis. Such portals have the main goal to facilitate the run of advanced models, through access to large-capacity computers or servers. In this study, we present the Analysis Portal (www.analysisportal.se), which is a part of the Swedish LifeWatch e-infrastructure for biodiversity research that combines a variety of Swedish web services to perform different kinds of dataprocessing.

**New information:**

For the first time, the Swedish Analysis Portal for integrated analysis of species occurrence data is described in detail. It was launched in 2013 and today, over 60 Million Swedish species observation records can be assessed, visualized and analyzed via the portal. Datasets can be assembled using sophisticated filtering tools, and combined with environmental and climatic data from a wide range of providers. Different validation tools, for example the official Swedish taxon concept database Dyntaxa, ensure high data quality. Results can be downloaded in different formats as maps, tables, diagrams and reports.

## Introduction

Biodiversity informatics is a young discipline. The term was probably firstly used in 1996 by J.L Edwards in an unpublished document by the OECD Working Group on Biological Informatics – 1^st^ meeting, notes on the draft agenda, Washington (see www.bgbm.org/BioDivInf/TheTermn.htm). It encompasses information techniques for all kinds of biodiversity information and a framework for data sharing to address questions in the field of conservation management and ecosystem services. Bisby 2000 and Edwards et al. 2000 presented one of the first accessible databases, like GBIF (www.gbif.org), Species2000/ITIS Catalogue of Life (www.sp2000.org), FishBase (www.fishbase.org) and others, that they called the ´silent digital revolution` and which became accessible on everybody’s desktop. The terms bioinformatics, eco-informatics and biodiversity informatics are frequently used today, but often lead to confusion and misunderstanding. Traditionally, bioinformatics is connected to the molecular biology, while eco-informatics are dealing with environmental factors, such as climate and abiotic measurements. Globally, the LTER-network (Long Term Ecological Research Network; http://www.lternet.edu), DataONE (Data Observation Network on Earth, https://www.dataone.org) and GEO BON (Group on Earth Observations Biodiversity Observation Network, http://geobon.org) are more focused on eco-informatics, while GBIF is more focused on biodiversity informatics. The term includes taxonomic databases as well as databases of species observation records and in a broader context even species distribution modelling.

Hardisty et al. 2013 realised the basic necessity to link existing biodiversity infrastructures to more effective systems, so-called electronic infrastructures (e-infrastructures), across sub-disciplines that can be shared by business people, citizen scientists, policy-makers and researchers. In general, research infrastructures (RIs) funded by the European Commission through the 7th Framework Programme (http://cordis.europa.eu/fp7/home_en.html), have the goal of offering research services, shaping scientific communities and supporting stakeholders in decision-making processes (ESFRI - European Strategy Forum on Research Infrastructure; http://ec.europa.eu/research/infrastructures/index_en.cfm?pg=esfri). One of numerous European RIs is LifeWatch.

The LifeWatch concept was begun to build an e-Science European infrastructure for biodiversity and ecosystem research in January 2011, where representatives from Hungary, Italy, the Netherlands, Romania and Spain signed the first Memorandum of Understanding. The idea was to construct a European research e-infrastructure project for biodiversity science and ecosystems research (www.lifewatch.eu). The LifeWatch architecture is described in detail elsewhere (Giddy et al. 2009, Basset and Los 2012, Fuentes and Fiore 2014, Gärdenfors et al. 2014). Some of the main tasks of the concept are 1) *Information and communication technology (ICT) support creating access to open interfaces hosting data and tools*, 2) *Virtual e-Laboratories where users can create and share their analyses and models*, and 3) *Capacity *building* and support through trainee workshops on the usefulness of and approach to all services*. A number of European countries (Belgium, Greece, The Netherlands, Italy, Romania, Portugal and Spain) have agreed to apply to the European Commission to form a European Research Infrastructure Consortium (ERIC). Some countries (Finland, France, Hungary, Slovakia, Slovenia and Sweden) have decided to initially connect to the ERIC as observers. Sweden was the first European country to begin to build a national LifeWatch e-infrastructure for biodiversity data.

The Swedish LifeWatch consortium was founded in 2010 and is a national collaboration between four universities (the Swedish University of Agricultural Sciences (SLU), the University of Gothenburg (GU), Lund University (LU) and Umeå University (UMU)), the Swedish Meteorological and Hydrological Institute (SMHI) and the Swedish Museum of Natural History (SMNH), including the Swedish GBIF node. The project is coordinated by the Swedish Species Information Centre (ArtDatabanken) at SLU and financed by the Swedish Research Council (VR) and the Swedish Environmental Protection Agency.

**Figure 1.**
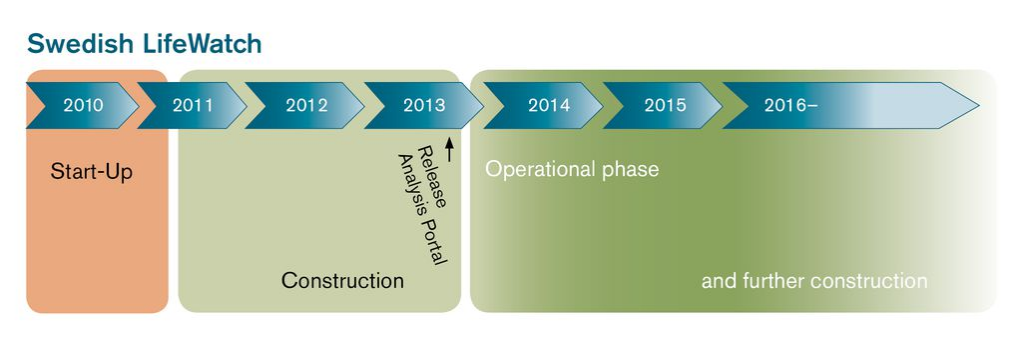
The time schedule of the Analysis Portal. From the start-up in 2011 over a three-years construction phase including its release, to a new operational phase and further construction.

The principal aim was to create a national web service oriented e-infrastructure for biodiversity data covering terrestrial, freshwater and marine habitats, and where all components are accessible through open web services (Gärdenfors et al. 2014). The idea was not to collect new data or create a new database, but provide one single entry point to existing databases in Sweden and enable free access to biodiversity and environmental data. The graphical user interface representing that entry point was named the Analysis Portal (www.analysisportal.se). This web application offers a range of analytical and visualisation services like a species richness grid map, time series and species observation maps. The first version of the Analysis Portal was put into operation in December 2013 (Fig. 1). Since the official release of the AP the total number of users has increased over the years (2014: 2,025, 2015: 6,231), similar the web page views, that rose from 52,050 (2014) to 77,382 (2015) (PV - Google analytics). Most of the users came from Sweden (2014: 84.5%, 2015: 55.2%).

This article describes the Analysis Portal (version 1.0.5821.25115, release date: 09/12/2015), which is a part of the Swedish national e-infrastructure of biodiversity data. In this software description, we refer to the current version named above. We describe the structure of this web application and the functionality of its different components. We also give a detailed presentation of all the web services the Analysis Portal is built upon (Supplementary materials 1-3).

## Project description

### Title

Swedish LifeWatch Analysis Portal - a national e-infrastructure for biodiversity research

### Study area description

**Figure 2.**
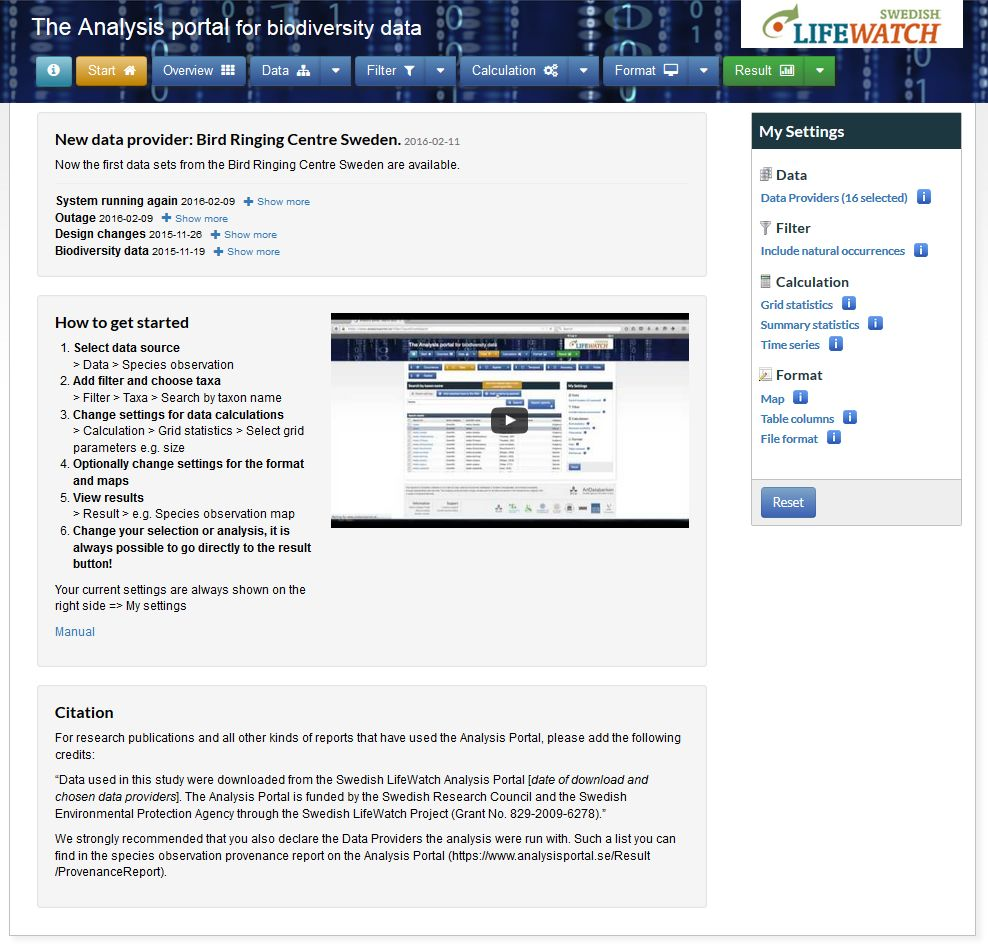
Start page of the Analysis portal.

The Analysis Portal (AP) (Fig. 2) is a part of the Swedish LifeWatch e-infrastructure for biodiversity research that combines a variety of Swedish web services to perform different kinds of data processing. One of the services, the Analysis Service, can process the species observation data that are harvested on daily bases from several data providers. The various kinds of functions that are used include calculations of species richness and numbers of observations for defined sets of taxa, polygons, grids or time series. The Analysis Service can also perform calculations on grid based summary statistics on specified WFS data layers. The inputs to the portal are species observation data, climate and environmental data as layers (WFS) and maps (WMS), drawn polygons and uploaded GeoJSON files. The user can visualise and download different kinds of data tables, maps, histograms and diagrams on species observation, species richness and time series of species records as output. The users of the AP are researchers and students, biologists and ecologists at authorities and consult companies as well as citizen scientists.

### Design description

**Data providers**

**Figure 3.**
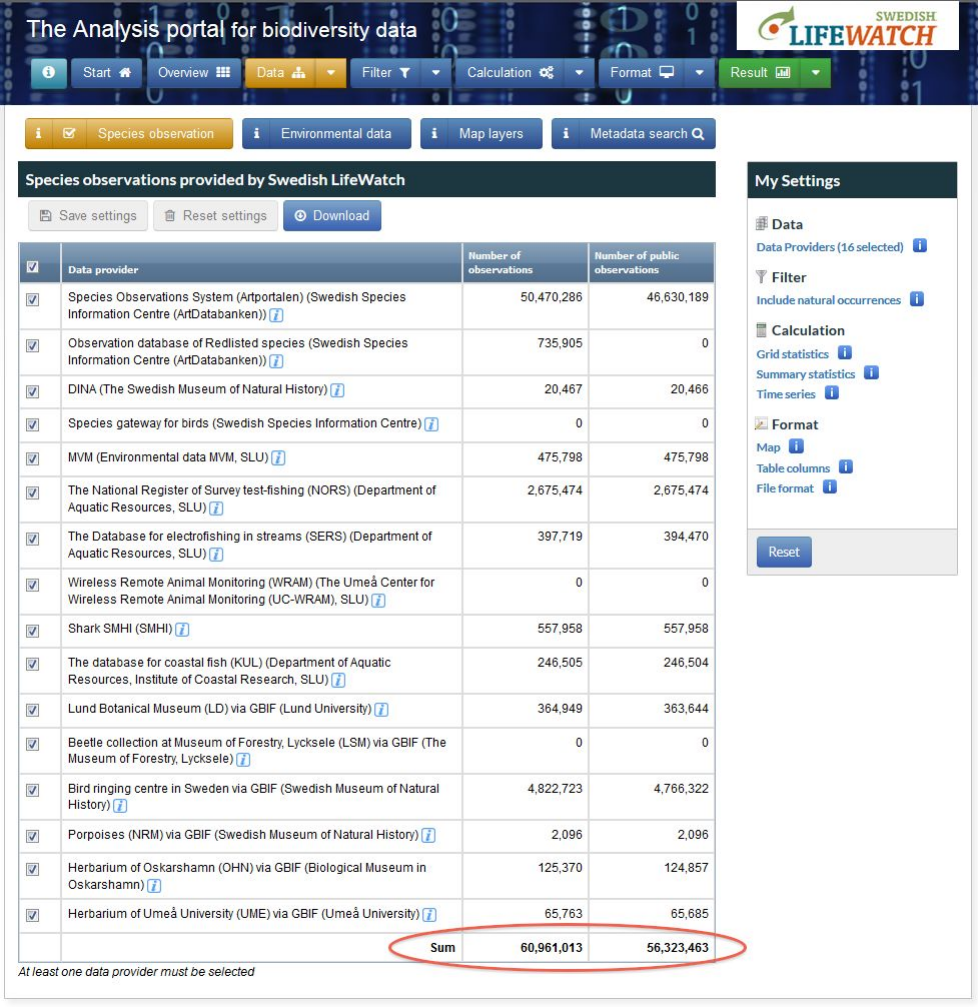
Overview of the connected national databases and their occurrence records to which the Analysis Portal offers access.

Numerous biodiversity occurrence databases are connected to the Analysis Portal via the Taxon Observation Service. The AP offers an entry node to all of these databases, currently providing access to around 60 million species observation records (Fig. 3).

The most comprehensive database on Swedish biodiversity data is the Swedish Species Observation System (http://www.artportalen.se) financed by the Swedish Environmental Protection Agency. Since 2000 it has been an internet-based, freely accessible reporting system and data repository for geo-referenced species observations of the major organisms, i.e. animals, plants and fungi. Today it provides more than 50,000,000 observations of 28,660 different species (Gärdenfors et al. 2014). Around 5,000,000 new observations are added to the database every year. The data come from citizen science, environmental monitoring programmes and research projects.

The Swedish Species Observation Centre also collaborates on the observation database of red-listed species with around 1 million occurrence data. The database contains only protected species and permission is required to have access to it. This is handled by the UserAdministration (see below: Data Management and Cleaning) where the rapporteur (http://www.artportalen.se/Home/TermsOfUse) and the user have to follow a certain policy formulated by the Swedish Species Information Centre (http://www.artdatabanken.se/verksamhet-och-uppdrag/arter-kunskapsinsamling/fynd-av-arter/skyddsklassade-arter/). In future, this data set will be integrated into the Swedish Species Observation System. A similar integration is planned for the freshwater fish database PIKE (Umeå University; http://www.emg.umu.se/english/research/research-projects/pike/?languageId=1#).

Another extensive database that is available via the AP is the national register of survey test-fishing (NORS, http://www.slu.se/en/departments/aquatic-resources/databases/national-register-of-survey-test-fishing-nors/) with more than 2,000,000 records on caught fish dating back to the 1950s. The standardized test-fishing with Nordic multi-mesh gillnets (12 mesh sizes) became a European standard (EN 14757) in 2005 and gives a good overview of species occurrence and abundance of fish fauna in lakes. For rivers, the Swedish Electrofishing RegiSter (SERS, http://www.slu.se/en/faculties/nl/about-the-faculty/departments/department-of-aquatic-resources/databases/database-for-testfishing-in-streams/) exists in Sweden. The database was begun in 1989 and most electric fishing is ordered by the Swedish Environmental Protection Agency and Swedish Agency for Marine and Water Management, that also finance part of data hosting. Both fish databases provide fairly good predictions of absence data. The coastal fish database (KUL, http://www.slu.se/en/departments/aquatic-resources/databases/database-for-coastal-fish-kul/) is currently in line to be linked to the AP. The data consist of fish catches with net and fyke net in Swedish coastal waters. The Department of Aquatic Resources at the Swedish University of Agricultural Sciences and the Swedish Agency for Marine and Water Management host the data.

Marine environmental monitoring data are represented by the Swedish Ocean Archive, the so-called SHARK database (www.sharkdata.se), with around 70,000 species occurrence data of zoo- and phytoplankton recorded by the Swedish Meteorological and Hydrological Institute (SMHI). Species observations on freshwater species are represented by the environmental data web service MVM (http://miljodata.slu.se/mvm/) linked with nearly 500,000 species occurrence data to the AP. The Department of Aquatic Sciences and Assessment at the Swedish University of Agricultural Sciences is the data host for inland waters and the MVM database containing data on national and regional monitoring of phytoplankton, benthic diatoms, macrophytes, zooplankton and benthic fauna.

Historical data sets are also linked to the AP through DINA (Digital Information system for Natural history collections; www.dina-web.net/naturarv/), a Swedish system for digitalization of natural history collections in Stockholm (Swedish Museum of Natural History, SMNH) and Gothenburg (Göteborg Natural History Museum, GNHH). At the moment, more than 20,000 objects with coordinates are linked to the AP, but digitalization will increase substantially in the near future.

By using the GBIF web service (www.gbif.org), several databases of different Swedish museums (e.g. the herbariums of the Oskarshamn Botanical Museum and Umeå University) are available with all geo-referenced records in the AP. Furthermore, the Swedish database on harbour porpoises (SMNH) (> 2,000) is linked to the AP. The Swedish bird ring centre´s (SMNH) species occurrence records, numbering approx. 5 million, has recently be linked.

Wireless Remote Animal Monitoring (WRAM) is a national e-infrastructure for automatic reception, long-time storage, sharing and analysing biotelemetry sensor data from animals like mammals, birds and fish (Dettki et al. 2014). Around 100 million data records come from biotelemetry sensors mounted on animal-borne tags and collars that record the location of the animal but also include data on physiology and behaviour. At the present the database is not accessible to the public, but the position records of the animals should be linked to the AP in the near future.

The long-time goal is to connect all national and regional Swedish databases to the AP via the Artportalen or directly to the Swedish Species Observation Service to make them accessible to researchers, policy-makers and citizen scientists through a single entry point. The number of occurrence reports in most of the databases increases substantially every year. Additionally, numerous Swedish databases at different host institutes are not connected today, for example national databases on seals, butterflies, hedgehog, moose and other game animals, as well as databases on invasive species, mussels, freshwater jellyfish and crayfish or the tree portal.

**Data Management and Cleaning**

The biodiversity data are structured in very different ways in the various databases that are connected to the Swedish LifeWatch e-infrastructure. In order to make all the data searchable at one single focal point, the data have to be restructured. Many of the data sources are sample-based and only contain partial information on taxon-specific occurrences. Darwin Terms (TDWG, http://rs.tdwg.org/dwc/terms/index.htm) provide a practical format suitable for sharing data on species observations and it is the main format adopted by the GBIF. For the Swedish LifeWatch e-infrastructure, a data model was constructed that could handle an infinite number of classes and data properties, including the set defined in Darwin Core (Wieczorek et al. 2012). This solution enables a flexible generic structure for handling all types of species observation data either strictly as Darwin Core or in any other way. So far we have succeeded in using the Darwin Core classes and their sets of properties with only a few minor additions (Swedish LifeWatch 2015; see SwedishSpeciesObservationService - Darwin Core). The SOAP (Simple Object Access Protocol) Service interface for this implementation is illustrated on this webpage.

The process of restructuring the data to fit Darwin Core and to enable a uniform search mechanism is supported by a set of web services. Each provider has published a web service where data can be retrieved according to the Darwin Core format. Regarding the taxonomic information only taxon id and the verbatim form of the scientific name, if available, is retrieved from the providers. Based on the taxon ids, the rest of the taxon-related information is retrieved directly from the Taxon Service, i.e. the SOAP-based web service behind the web application Dyntaxa (www.Dyntaxa.se). This solution ensures that the taxon-related search mechanisms provided by the Swedish LifeWatch focal point services are always based on taxon hierarchies and naming that is up to date irrespective of the status of local providers. As Dyntaxa is used when administrating changes of taxon concepts, the Taxon Service can provide the necessary information to resolve effects of lumps and splits in a correct manner.

A specific service named the Species Observation Harvest Service has been constructed in order to perform the harvesting and data processing necessary for compiling a unified dataset fulfilling all the requirements set by Swedish LifeWatch based on what is retrieved from each provider. The Harvest Service has methods to start and cancel harvests of single providers. To connect a new data provider service, one only needs to specify a mapping protocol that determines which field(s) in the Darwin Core set should correspond to the properties in the provider´s interface. Sometimes, data from the provider needs processing and sometimes default values are set for a single provider for certain fields.

**Figure 4.**
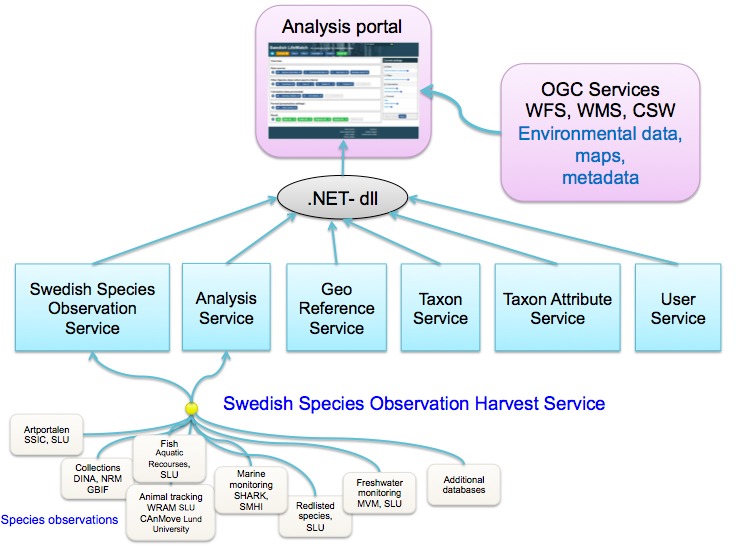
Overview of the web services used in the AP. The core web services (SOAP) are shown in blue boxes. Two of them handle species observations harvested from a number of data provider services (white boxes). Environmental data, maps and metadata of different kinds are connected to OGC services (WFS, WMS, CSW).

The Harvest Service cleans the data in various ways by checking the content of each data field. If a data field contains values that do not correspond to the rules specified, the record is discarded and logged as erroneous. The error logs is a useful source of information in the process of increasing the quality of the harvesting mechanisms (Fig. 4).

The user administration provides the user options for personalized settings and allows certain search enquiries to be saved and reused. The login is not necessary. The same user administration system is used for the Swedish Species Observation System (Artportalen), Dyntaxa and the AP. For each user, over 90% of observations are publicly accessible; exceptions exist only for a few sensitive species (http://www.artdatabanken.se/media/2157/skyddsklassade-arter-artdatabanken-150505.pdf). Access to sensitive species is subject to certain user requirements and permissions are handled via the user administration system (Swedish LifeWatch 2015; see Applications - UserAdmin).

**Systems architecture**

The AP is a web application that has been constructed according to the Model-View-Controller (MVC) design pattern using ASP.NET MVC. The application is not connected directly to any database; all data retrieval mechanisms, statistical calculations, and aggregations are instead performed by various web services (Fig. 4). This implies that functionality presented in the AP is not uniquely provided by that software. In fact, the same functionality may in the future be incorporated in a vast array of applications specially designed for specific user groups. The number of applications that utilize the same services in different ways is increasing. To facilitate this development process, a reusable code package (.NET-dll in Fig. 4) can be downloaded from the SLW home page (Swedish LifeWatch 2015). This code represents an object-orientated data model that handles all data types handled by the core web services in Swedish LifeWatch.

Another implication of the service-oriented architecture is that the software is only loosely dependent on the underlying data sources. It should be possible to connect the AP to another set of services providing data from another region. Actually, if for example the GBIF web services were to be complemented with corresponding functionality, the AP could work on a global scale without much reconfiguration. This is an interesting idea indeed, as some of the most powerful methods provided by the AP or rather its underlying web services, would in many cases require uploads of hundreds of millions of records in order to perform the statistical processing based on the functions available in the RESTful web service API currently provided by GBIF. This is of course not really a feasible solution. However, by adding a couple of analytical functions (Table 1, Suppl. material 1) which can retrieve spatial and temporal summary statistics of different kinds (e.g. counts of occurrences and species) and a somewhat greater set of search criteria (Table 2, Suppl. material 2) to the existing GBIF web service API, it would be a simple task to replace the current web service connections of the AP with connections to the GBIF web service API.

**Services**

The AP currently uses three types of service. Two belong to the suite of standardized OGC (Open Geospatial Consortium http://www.opengeospatial.org) services, i.e. the Web Feature Service (WFS), which is suitable for retrieving geo-referenced data of any kind, and the Web Map Service (WMS), which provides raster representations of the data. The third type of web service that the AP uses is a set of very specific SOAP services that are considered to be the core services in the Swedish LifeWatch e-infrastructure for biodiversity data.

**Figure 5.**
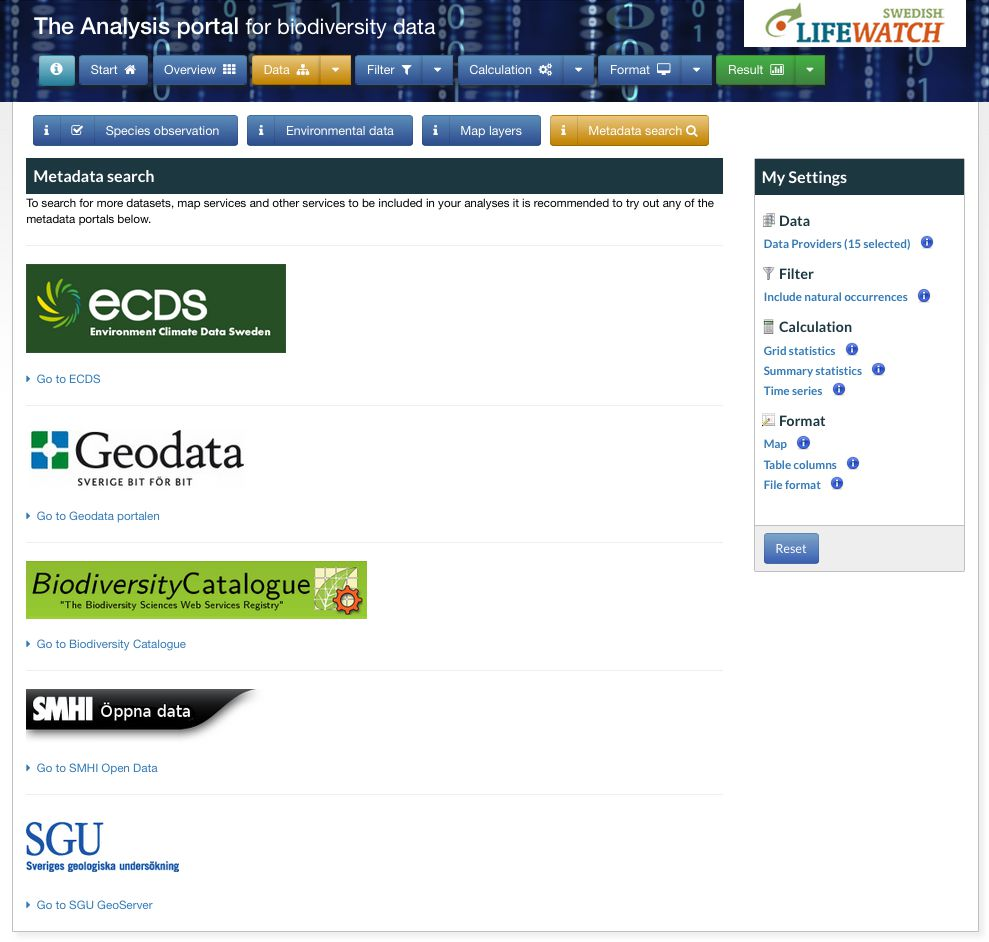
Overview of linked metadata search functions of the AP.

The generic REST-based interoperability of the OGC Services makes them easy to integrate dynamically in web applications using, for example, Open Layers. In the AP, this was done mainly for the purpose of giving the users the flexibility of adding whatever environmental or climatic data and maps that are made available by data providers all over the world. To find detailed information about these services, we recommend a set of Swedish metadata portals (Environment Climate Data Sweden, ECDS; the Geodata Portal; the Swedish Metereological and Hydrological Institute, SMHI Open Data Catalogue; Geological Survey of Sweden, SGU Open Data Catalogue) (Fig. 5). All these portals handle metadata according to the OGC Catalogue Service Standard.

The medium-term ambition is to implement support for all major OGC services in the AP, including direct searches for data using the Catalogue Services mentioned above. Currently, the AP only supports OGC for connecting environmental data via the OGC Web Feature Service (WFS) and as background maps using the OGC Web Map Services (WMS). Most available OGC types of services that are relevant for the LifeWatch community are provided by organizations that do not belong to the Swedish LifeWatch Consortium and the services have been published for quite different reasons. Many of them have been published due to the INSPIRE directive (http://inspire.ec.europa.eu). Swedish LifeWatch, however, does also publish some data, e.g. WFS. One of the layers SLW provides consists of all public species observations as Darwin Core (http://slwgeo.artdata.slu.se:8080/geoserver/web/?wicket:bookmarkablePage=:org.geoserver.web.demo.MapPreviewPage).

As the Core Services are totally essential for the functionality in the AP, we describe each of them briefly below. We will also explain in detail the web service functions that are actually used in the current version of the AP in Table 1 (Suppl. material 1) and the user manual (Suppl. material 3). As the core services include more functions than listed in Table 1 we recommend consulting the full interface documentation for more information (ArtDatabankenSOA https://Doc.ArtDatabankenSOA.se).

In general, there exist six main services:

1) *User Service*

Url: https://user.artdatabankensoa.se/UserService.svc?wsdl​

This is the main service used by the web application UserAdmin (https://UserAdmin.ArtDatabanken.se), which is the main administration tool for authorization within the Swedish LifeWatch. The service supports generic construction of application specific authority objects and user roles which can be used in order to regulate which users are allowed to retrieve or edit data of different kinds. The service supports regulation of data handling based on taxonomic, temporal and spatial specifications. It also supports delegation of authorization in a rather simple and effective way, making it possible, for example, for certain members of the Swedish County Administrations to authorize their own trusted employees to handle sensitive data within their geographical region.

The User Service is used by all other core services for authentication and in order to check the user’s roles and authorizations.

2) *Taxon Service*

Url: https://taxon.artdatabankensoa.se/TaxonService.svc?wsdl​

This is the main service used by the web application Dyntaxa (www.dyntaxa.se) to search and present taxonomic information on practically all multicellular taxa naturally occurring in Sweden. This service also provides all methods utilized by Dyntaxa for all editing of the content in the Swedish Taxonomic Database. Taxon Service provides the infrastructure with globally unique identifiers of all the taxonomic concepts that are handled by the core web services within Swedish LifeWatch. The Dyntaxa taxon id, which is the name of the taxon concept id in Dyntaxa, is the main link between the different data types essential for biodiversity in Sweden, such as species observations, taxon names and hierarchies, taxon traits and other attributes, as well as information on legislation and Red List status.

3) *Taxon Attribute Service*

Url: https://taxonattribute.artdatabankensoa.se/TaxonAttributeService.svc?wsdl​

This service has the potential to handle all generalizations about taxa including habitat and substrate preferences and usage, interspecific interactions, life history traits, threats, Red List classification, and legislation. It handles more than 2,000 factors that are evaluated in relation to the Swedish taxa provided by the Taxon Service. In the scope of LifeWatch, the service is mainly used for retrieving taxon lists determined by different factors or combinations of factors and taxonomic hierarchies.

4) *Geo Reference Service*

Url: https://georeference.artdatabankensoa.se/GeoReferenceService.svc?wsdl​

This service provides information on existing Swedish regions of different kinds, e.g. counties and municipalities.

5) *Swedish Species Observation Service*

Url: https://swedishspeciesobservation.artdatabankensoa.se/SwedishSpeciesObservationService.svc?wsdl​

This service constitutes the main focal point of Swedish biodiversity data in terms of species observations and occurrence data. It provides a couple of methods that can be used in order to retrieve species observations originating from several data sources, e.g. Darwin Core records. In addition to the methods (9-16) listed in Table 1 Suppl. material 1, it has methods that support incremental harvesting of observation data. One of these methods is used by the GBIF in order to harvest public occurrence data from Sweden.

6) *Analysis Service*

Url: https://analysis.artdatabankensoa.se/AnalysisService.svc?wsdl

In contrast to the other services, this service does not handle a particular type of data. Instead, it is dedicated for all sorts of data processing or data retrieval tasks that involve transformations of data types from their basic representation to something else (Swedish LifeWatch 2015; see Analysis Service). Most functions are related to species observations in one way or another and many of these functions are controlled using the same search criteria (WebSpeciesObservationSearchCriteria) as those used when retrieving species observations or Darwin Core records from the Swedish Species Observation Service. Instead of the record *per se* the Analysis Service delivers summary statistics of species observations aggregated taxonomically (Functions no. 19-20, Table 1), temporally (no. 21, Table 1) or spatially (no. 22-23, Table 1) (Suppl. material 1).

The Analysis Service also includes a number of processing methods that act on data from OGC WFS. These methods can be used for calculating grid statistics based on the features in a specified data layer (Functions no. 25-26, Table 1, Suppl. material 1).

More information on the usage of the SLW Core Service can be obtained from the Swedish LifeWatch homepages (SLW Data Management, 2015 or www.servicecentrelifewatch.eu/products).

**Portal functionalities**

The AP has numerous functionalities. Species occurrence records can, for example, be filtered by present and absence data, or after taxa name, polygon, time and red listed category. Different environmental layers (WMS, WFS) can be uploaded to the AP and combined with species observation records. All functionalities of the current version are described in the user manual (Suppl. material 3). The user manual also refers to Table 1 (Core Web Service Methods, Suppl. material 1) and Table 2 (List of criteria that can be specified when searching for species observations, Suppl. material 2) where more technical details are available and linked to each of the described functionality.

**Users**

**Figure 6.**
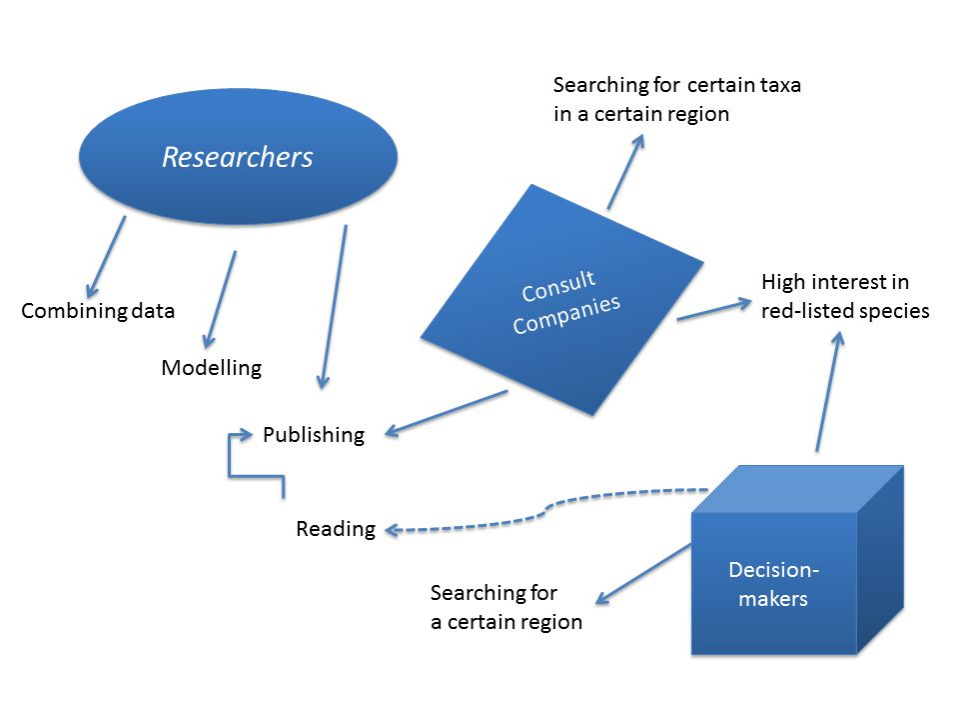
The three different user groups and their connection: 1) Researchers that combine data from the portal with their own data, running models and publishing the results; 2) Consulting companies getting the order to analyse a certain area for environmental effects or have to inventorise red-listed species. Beside writing reports, scientific publishing can be found as well (e.g. Helldin et al. 2015). Decision-makers are interested in following up the distribution of red-listed species or the species diversity in a certain region. They have to taken into account the published work of the other two user groups.

At present, the AP has three different kinds of main users: researchers, biologists at the county/municipality/other administrations and consulting companies (Fig. 6). Researchers mostly download data sets for further analysis to work with in their own software environments (e.g. QGIS, R, Taverna). More and more data sets accessible through data centres and online portals are being used in science and published, e.g. in the context of species distribution modelling (e.g. Bellamy and Altringham 2015, Leidenberger et al. 2015). Although the catalogue of WMF layers often helps PhD students, for example, to get a first overview of available layers and their providers in Sweden and find the desired layer(s) and resolution for the analysis.

Ecologists and biologists working for an authority or a consulting company, working with environmental impact analysis and decision-making processes, often use the taxon id filter function to download, for example, red-listed species in a certain area (polygon filter). User administration handles access to protected species, where different authorities have access to several existing protection levels (1-5) of the species, depending on their responsibilities in the decision-making processes.

Today, 2 years since the release of the AP, it is not clear to what extent citizen scientists use the portal. From Artportalen, an important Swedish data provider, it is known that numerous ornithologists, members of entomological and botanical associations report to and use the database to compare and share local species information. In future, it is hoped that these user groups will join the company of the AP to run simple analyses for their target species.

In the future, many analyses can be run through the portal and the use of the available data in the AP may lead to further ideas, observations and investigations of species distribution patterns. Gärdenfors et al. 2014 give some examples of how a user can investigate biodiversity of species with the help of the AP.

On-going research that use the AP includes, for example, phenology studies on birds and effects of habitat restorations on wetland bird trends. Another study is attempting to identify forest regions based on species composition in order to make regionalized conservation recommendations. Studies have already been published that have been done on the SLW infrastructure (http://www.svenskalifewatch.se/sv/publicerat/). One recently published study deals with the identification of candidate responsibility species that are of particular interest in connection with the planning of road and railway constructions (Helldin et al. 2015). Another study presents an algorithm to display the lack and bias in sampling efforts through so-called ignorance maps (Ruete 2015).

**Outlook**

The Analysis Portal described in this paper is a first national step towards making biodiversity data free available to scientists, companies and decision-makers dealing with questions of and related to nature conservation management (Fig. 6). The SLW e-infrastructure of web services is used in different contexts and several user backgrounds have varying needs for such a portal. The first version of the AP tried to combine as much functions as possible on biodiversity data and was built on the basis of users` feedback.

The AP will continue to be developed in response to users` feedbacks and requests. An interesting idea would be to adjust the AP to function on a global scale instead of only for Sweden. Practically, this could, for example, be done by complementing the GBIF web service with the functionalities listed in Table 1, Suppl. material 1.

One vision of Swedish LifeWatch is that all Swedish biodiversity data, or at least those that are produced with taxpayers` money, are freely accessible via a portal like the AP that is based on numerous web services. Today, the technical know-how is available, but co-operation between all institutes, universities, scientists and other responsible people is still under development. Perhaps it also needs a generation shift, where younger scientists grow up with a better data-sharing mentality. Bio(diversity)informatics is in demand to be taught more at university level (Severin 2011). Trainee workshops for user groups outside the universities and support for the use of biodiversity e-infrastructure in general are also necessary and has to increase in the near future.

Following the basic concept of LifeWatch, Swedish LifeWatch is actively working on capacity building, giving trainee workshops and support, and building up a Nordic LifeWatch co-operation between the Scandinavian countries to combine existing e-infrastructures into a Nordic network, similar to the AP. Long-term perspectives might be to create related e-infrastructures on biodiversity data on the European level. Canhos et al. 2015 realised that biodiversity e-infrastructures are important frameworks for national and international collaborations as well as key facilities in shared data and knowledge between scientific development, policies and decision support systems.

Species do not stop in front of political borders and successful biodiversity research and management therefore need more free access and availability of data on a national and international level. This can push collaboration forward and will help us solve prospective challenges in the field of nature conservation management in future.

### Funding

Swedish LifeWatch is funded by the Swedish government through the Environmental Protection Agency and the Swedish Research Council (grant no. 829-2009-6278).

## Web location (URIs)

Homepage: https://www.analysisportal.se/

## Usage rights

### Use license

Creative Commons Public Domain Waiver (CC-Zero)

## Supplementary Material

Supplementary material 1 Core Web Service Methods Data type: pdf
Brief description: A list of all core web service methods used in the AP. Here, the published methods name includes the name of the SOAP service. The functional description gives details about what can be arbieved with each method.
File: oo_82227.pdf Leidenberger et al. 2016

Supplementary material 2 List of criteria that can be specified when searching for species observations Data type: pdf
Brief description: List of criteria that can be specified when searching for species observations in the Swedish LifeWatch e-infrastructure core web services, i.e. the Analysis Service and the Swedish Species Observation Service.
File: oo_81905.pdf Leidenberger et al. 2016

Supplementary material 3 The Analysis Portal - user manual Data type: pdf
Brief description: User manual - portal functionalities.
File: oo_82233.pdf Leidenberger et al. 2016
