# Supplementary material for: The Analysis Portal and the Swedish LifeWatch e-infrastructure for biodiversity research
Source: Biodivers Data J. 2016 Mar 23;(4):e7644. doi: 10.3897/BDJ.4.e7644 (PMC4822057; doi:10.3897/BDJ.4.e7644)
Supplement: Supplementary material 1 — Core Web Service Methods [file biodiversity_data_journal-4-e7644-s001.pdf]

Table 1: A list of all core web service methods used in the Analysis Portal. Here, the published method name includes the name of the SOAP service. The functional description gives details about what can be achieved with each method.

**Method**	**Functional description**
**1**. UserService: Login	A user must login to a web service before any other methods in the web service are called. If several web services in ArtDatabankenSOA are used, the user must log in to each web service separately. Login is made by a call to the Login method, which on successful login returns a security token. This security token must be kept by the client application and is provided in further calls to the web service. When the user has finished using the web service, a call to the Logout method should be made.
**2**. TaxonService: GetTaxonNamesBySearchCriteria	Returns a list of taxon names that matches the taxon name search criteria. Each name record holds information on name status, name type and the associated taxon concept with its recommended scientific name and rank (taxon category, e.g. Species, Genus, Family, Order etc.).
**3**. TaxonService: GetTaxaBySearchCriteria	Returns a list of taxa that match the taxon search criteria.
**4**. TaxonService: GetTaxonChange	Get a list of changes made in the taxon tree. Current version returns changes regarding: - new taxon - new/edited taxon name (scientific + common) – lump/split events – taxon category changes.
**5**. TaxonAttributeService: GetTaxaBySearchCriteria	Get taxa that match species fact search criteria.

**6**. TaxonAttributeService: GetFactorTreesBySearchCriteria	Get information about factor trees that matches the factor tree search criteria.
**7**. GeoReferenceService: GetRegionsByCategories	Get region categories. All region categories are returned if the parameter countryIsoCode is not specified.
**8**. GeoReferenceService: GetRegionCategories	Get regions related to specified region categories. A region category could be Swedish Municipalities, Counties or Landscape provinces.
**9**. SwedishSpeciesObservationService: GetSpeciesObservationDataProviders	Returns a list of all data providers of species observations currently connected to the Swedish LifeWatch infrastructure. The list contains the name, description, contact information of each provider plus total counts of public and non-public data records. Each provider´s metadata is read from the database. Numbers of observations are calculated once per day and only include occurrences. Observations where the reported taxon was not present are excluded from these summary statistics.
**10**. SwedishSpeciesObservationService: GetSpeciesObservationFieldDescriptions	This method provides information about all species observation data fields handled currently by Swedish LifeWatch. Indications of whether or not the field represents an accepted DarwinCore term according to TDWG and whether or not it is a searchable field. Each field has a description of its general content and a list of data provider specific descriptions of how the field has been mapped to original data or other aspects of how the filed has been implemented.

**11**. SwedishSpeciesObservationService: GetDarwinCoreByIds	Get requested species observations. Scope is restricted to those observations to which the user has access rights. Species observations are returned in a format that is compatible with Darwin Core. A maximum of 100,000 observations with information can be retrieved in one call.
**12**. SwedishSpeciesObservationService: GetDarwinCoreBySearchCriteria	Returns a list of species observation records with a specific set of columns defined by Darwin Core (TDWG) plus a few additional fields specifically needed by many Swedish users. The actual records retrieved by this method are determined both by the specified search criteria and the selected user role. Depending on which authorizations have been attached to the role, the response may also include non-public records. A maximum of 25,000 Darwin Core records can be retrieved in one call. If the search criteria match more records than this, ids for the remaining records are returned (a maximum of 1, 000,000 ids can be retrieved in one cell).
**13**. SwedishSpeciesObservationService: GetDarwinCoreBySearchCriteriaPage	Get information about species observations that match the search criteria. This method provides paging functionality of the result. The maximum page size is 10,000 species observations. Scope is restricted to those observations to which the user has access rights. Species observations are returned in a format that is compatible with Darwin Core.
**14**. SwedishSpeciesObservationService: GetSpeciesObservationsByIds	Get requested species observations. Scope is restricted to those observations the user has access rights. Species observations are returned in a format that is compatible with Darwin Core. A maximum of 25,000 observations with information can be retrieved in one call.

**15**. SwedishSpeciesObservationService: GetSpeciesObservationsBySearchCriteria	Get information about species observations that match the search criteria. A maximum of 25,000 observations with information can be retrieved in one call. A maximum of 1,000,000 observation ids can be retrieved in one call.
**16**. SwedishSpeciesObservationService: GetSpeciesObservationsBySearchCriteriaPage	Get information about species observations that match the search criteria. This method provides paging functionality of the result. The maximum page size is 10,000 species observations. Scope is restricted to those observations to which the user has access rights.
**17**. AnalysisService: GetSpeciesObservationCountBySpeciesObservationSearchCriteria	Returns the number of species observations that meet the search criteria and that fit the user´s authorization specifications.
**18**. AnalysisService: GetSpeciesCountBySpeciesObservationSearchCriteria	Returns both the number of species and the number of species observations that meet the search criteria and that fit the user´s authorization specifications. Species is here calculated as the distinct number of unique taxon Ids representing taxon concepts with the rank (=taxon category) equal to ‘species’. The algorithm takes the taxon hierarchy into account, which implies that observations of subtaxa of rank than ‘species’ are counted as their parent species.
**19**. AnalysisService: GetTaxaBySpeciesObservationSearchCriteria	This method provides a list of taxa which correspond to the taxa associated to the species observations that meet the search criteria and fit the user´s authorization specifications. It lists all reported taxa regardless of rank.

**20**. AnalysisService: GetTaxaWithSpeciesObservationCountsBySpeciesObservationSearchCriteria	This method returns a list of observed taxa with taxon specific numbers of species observations that meet the search criteria and fit the user´s authorization specifications.
**21**. AnalysisService: GetTimeSpeciesObservationCountsBySpeciesObservationSearchCriteria	This method returns a list of time step specific counts of species observations that meet the search criteria and fit the user´s authorization specifications. Time step length and periodicity can be specified. Only observations where both the start and end observation time is within the time frame specified for the time step will be counted.
**22**. AnalysisService: GetGridSpeciesObservationCountsBySpeciesObservationSearchCriteria	This method provides a list of grid cell specific counts of all the species observations that fulfil the search criteria and fit the user´s authorization specifications. Selecting a coordinate system and width of grid cells specifies the grid. All grid cell counts are retrieved from the database in a single call. The procedure use a group by method based on the floor values of the coordinates the observations represented in the selected coordinate system. This method enables free selection of grid sizes specified as any integer value (1-∞).
**23**. AnalysisService: GetGridSpeciesCountsBySpeciesObservationSearchCriteria	This method provides a list of grid cell specific summary statistics that are calculated from all the species observations that fulfil the search criteria and fit the user´s authorization specifications. The summary statistics include both, species count and total number of observations within each grid cell. Selecting a coordinate system and width of grid cells specifies the grid.

**24**. AnalysisService: GetProvenancesBySearchCriteria	Using the same search criteria as for all other core web service methods listed here that handle species observations, this method provides information about the provenance of the data. The response lists the owner(s), recorder(s) and data provider(s) of the observations that fit the search criteria together with counts of data records.
**25**. AnalysisService: GetGridFeatureStatistics	This method provides a list of grid cell specific summary statistics that are calculated from a specified web feature service layer (OGC WFS). The layer is specified by its URL. The set of grid cells is determined by selecting a coordinate system, grid cell size and a bounding box. The returned statistics depend on the geometry type of the WFS layer. For all types, counts of features within each cell are returned. For lines, the sum of lengths is also provided. For polygons, the total area of features and the total length of all boundaries within each cell are provided. The calculation of summary statistics is performed directly on the list of features retrieved from the WFS based on the specified URL using the GetFeatures method (http://www.ogcnetwork.net/node/178). The counts of feature are simply the number of features that are at least partly is represented within the quadratic grid cell. The length is calculated as the sum of the lengths of all the parts of all lines or edges (in case of polygons) that exist within the grid cell. The area is calculated as the total area of features existing within the grid cell.

**26**. AnalysisService: GetGridFeatureStatisticsCombinedWithSpeciesObservationCounts	Gets the grid cell feature statistics combined with both species observation and species counts. This method provides a list of grid cell specific summary statistics based on specified grid properties, URL to an OGC WFS Layer and a set of species observation search criteria. The resulting summary statistics contain grid cell specific counts of observations, species, and features and depending on feature type also total feature area (polygons), length of edge (polygons) or sum of feature lengths (lines). The grid cell specific summary statistics on species observations are calculated in the same way as described for method 23. The corresponding feature statistics are calculated as in method 25. The resulting list of summary statistics is the union of all cells, which contain either at least one observation or at least one feature.
