# Supplementary material for: The Analysis Portal and the Swedish LifeWatch e-infrastructure for biodiversity research
Source: Biodivers Data J. 2016 Mar 23;(4):e7644. doi: 10.3897/BDJ.4.e7644 (PMC4822057; doi:10.3897/BDJ.4.e7644)
Supplement: Supplementary material 2 — List of criteria that can be specified when searching for species observations [file biodiversity_data_journal-4-e7644-s002.pdf]

Table 2: List of criteria that can be specified when searching for species observations in the Swedish LifeWatch e-infrastructure core web services, i.e. the Analysis Service and the Swedish Species Observation Service.

**Name**	**Description**
**1**. TaxonIds	Limit search to taxa with specified ids. Ids for taxon are defined in web application Dyntaxa (www.Dyntaxa.se). Species observations made on sub taxa to specified taxa are also included in returned species observations.
**2**. ValidationStatusIds	Return only observations with specified validation status. This property is currently not in use.
**3**. IncludeRedListCategories	Search observations based on taxa that is red listed in any of the specified red list categories. If property IncludeRedListCategories is not empty all taxa that is red listed in any of the specified red list categories are added to property TaxonIds by the web service. Provided red list categories must be among the values DD (data deficient), RE (regionally extinct), CR (critically endangered), EN (endangered), VU (vulnerable) or NT (near threatened) in the enum RedListCategory. The other values in enumeration RedListCategory are not valid search criteria.
**4**. IncludeRedlistedTaxa	Search observations based on red listed taxa. If property IncludeRedListedTaxa is set to true all red listed taxa is added to property TaxonIds by the web service.
**5**. MaxProtectionLevel	Only observations that has a protection level that is equal to or lower than the value of this property are included in the result. 1 is the lowest possible value.
**6**. MinProtectionLevel	Only observations that has a protection level that is equal to or higher than the value of this property are included in the result. 1 is the lowest possible value.
**7**. SpeciesActivityIds	Limit returned observations based on species activities. Use method GetSpeciesActivities in web service SwedishSpeciesObservationService to retrieve currently used species activities. This property is currently not used.

**8**. BirdNestActivityLimit	Limit returned observations based on bird nest activity level. Only bird observations in Artportalen are affected by this search criteria. Only bird observations with the specified bird nest activity level or stronger may be returned. Observation of other organism groups (not birds) are not affected by this search criteria. Use method GetBirdNestActivities in web service SwedishSpeciesObservationService to retrieve currently used bird nest activities. Property BirdNestActivityLimit should be set to the Id value of the selected bird nest activity. Set property IsBirdNestActivityLimitSpecified to true if this property is used.
**9**. IsNaturalOccurrence	Restrict search based on if a positive observation is natural or not. Property IsNaturalOccurrenceSpecified indicates if property IsNaturalOccurrence should be used or not.
**10**. IncludeNeverFoundObservations	This property indicates whether to search for never found observations. "Never found observations" is an observation that says that the specified species was not found in a location deemed appropriate for the species.
**11**. IncludeNotRediscoveredObservations	This property indicates whether to search for not rediscovered observations. "Not rediscovered observations" is an observation that says that the specified species was not found in a location where it has previously been observed.
**12**. IncludePositiveObservations	This property indicates whether to search for positive observations. "Positive observations" are normal observations indicating that a species has been seen at a specified location.
**13**. FieldSearchCriteria	Limit search based on values for any data that is related to species observations.
**14**. LocalityNameSearchString	String search criteria to match with locality names. Exactly one string compare operator must be specified.
**15**. BoundingBox	Limit returned observations to specified bounding box. Currently only two-dimensional bounding boxes can be used.

**16**. Polygons	Search observations that are inside specified polygons.
**17**. RegionGuids	Limit search to specified regions. Regions are defined by GeoReferenceService.
**18**. RegionLogicalOperator	Specify how regions, polygons or bounding box should be logically combined when species observations are searched. The logical operator OR are used between spatial geometries. This property is currently not used.
**19**. Accuracy	Requested minimum accuracy of the coordinates. Species observations with bad accuracy (i.e. higher accuracy value) will not be included in the search result. The unit is meters. Use values equal to or greater than zero. Parameter IsAccuracySpecified must be set to true if property Accuracy should be used.
**20**. IsAccuracyConsidered	Indicates if species observations that are outside geographic area (e.g. bounding box, polygons or regions) but close enough when accuracy of observation are considered should be included in the result. This property has no impact on returned information if no geographic area is involved in the search.
**21**. IsAccuracySpecified	Indicates if property Accuracy has been set.
**22**. ObservationDateTime	Search observations based on observation date and time. Not all functionality in WebDateTimeSearchCriteria are implemented. Current restrictions: Only Days are handled if Accuracy has been specified.
**23**. ObserverIds	Search observations based on observers. Observer id corresponds to person id in the user service. Observations that are not related to persons defined in user service will not match this search criteria. This property is currently not used.
**24**. ObserverSearchString	String search criteria to match with observer names. Exactly one string compare operator must be specified.

**25**. ProjectGuids	Get observations related to specified projects (projects corresponds to "syfte" in Artportalen 1). This property is currently not used.
**26**. ReportedDateTime	Search observations based on reported date and time. Not all functionality in WebDateTimeSearchCriteria are implemented. Current restrictions: Only Days are handled if Accuracy has been specified.
**27.** ChangeDateTime	Search observations based on date and time when the observation was last changed. Not all functionality in WebDateTimeSearchCriteria are implemented. Current restrictions: Only Days are handled if Accuracy has been specified.
**28.** DataProviderGuids	List of data sources to use in the search. All data sources are used if no specific data sources are provided.
