# Supplementary material for: The Analysis Portal and the Swedish LifeWatch e-infrastructure for biodiversity research
Source: Biodivers Data J. 2016 Mar 23;(4):e7644. doi: 10.3897/BDJ.4.e7644 (PMC4822057; doi:10.3897/BDJ.4.e7644)
Supplement: Supplementary material 3 — The Analysis Portal - user manual [file biodiversity_data_journal-4-e7644-s003.pdf]

User Manual - Portal functionalities

In this part, a description of the different functionalities of the Analysis portal (AP) is given.

The start page gives an overview of the functionalities of the portal (Fig. S1). The eight main headings are information, start page, overview, data, filter (species observation search criteria), calculation (data processing), format (presentation and export settings) and result (maps, tables, diagrams, reports and downloads). The information button in the headline is linked to the Swedish LifeWatch homepage, where the user will find facts and background on the AP and news on the Swedish LifeWatch project in general (http://www.svenskalifewatch.se/en/about/analysis-portal/). The start page informs the user about current news on the AP, a short introduction including a video clip, a manual and how to cite the e-infrastructure. On the overview page, each of the main headings has an information button explaining the available functions of each heading, which is divided into further categories that can be chosen for the analysis. The headings under the headline "The Analysis Portal for biodiversity data" are visible during the entire analysis and can be chosen through an interactive mode.

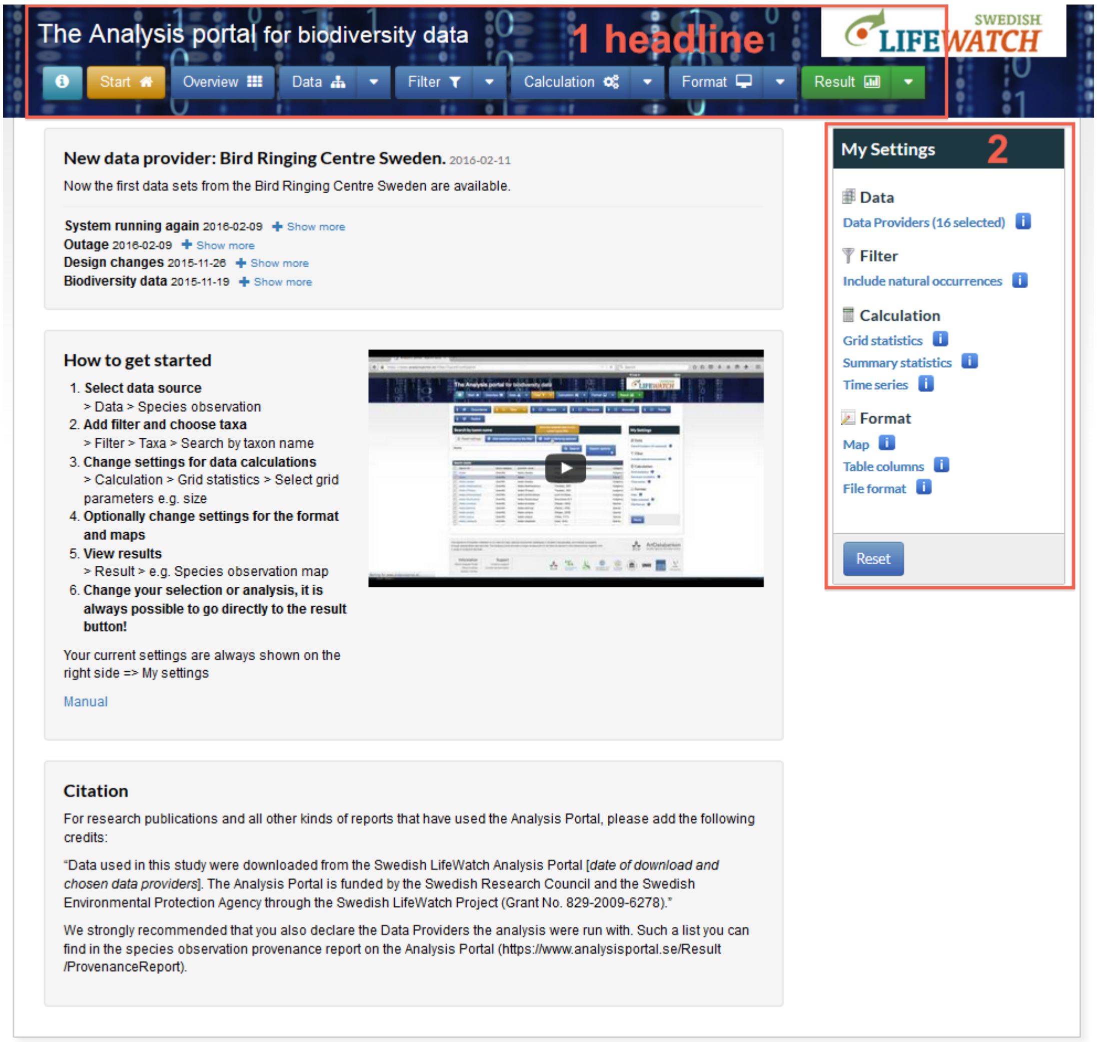

Figure S1: Start page of the AP: 1) the headline with the title and main headers, 2) *My settings* show the user the selected data providers, filter, statistics and formats.

On the right-hand of the portals interface the current settings (*My settings*) are shown for the user and give an overview about the selected data providers (by default: all are selected), filter(s) (by default: presence - include natural occurrence), calculations like grid statistics (by default: grid size 10x10 km, SWEREF99 TM coordinate system (EPSG:3006)), summary statistics (by default: calculations for number of observations and number of species) and time series (by default: month of the year) (Fig. S1). The format functions contain the map settings (by default: Google Web Mercator (EPSG:3857)), table settings (by default: minimum column set) and the file format (by default: comma separated and Excel .xlsx). Behind the settings, information buttons explain through a pop up window the different functions that were chosen in each category for the analysis. The current settings can also be reset.

At the bottom of the page, different links are provided to the AP, cookies, version number, but also to the support (e-mail contact), the current settings status and the logotypes of the SLW consortium´s logotype.

1. Data

Species observation

The user can start the analysis by choosing the data providers (> Data – Species observation – Data provider). Automatically, all data providers are chosen but can be deselected. At least one data provider must be selected in each run. If changes/settings are made on the AP´s interface, these must be saved before the analysis is run. Information about the data and data provider is given in the paragraph Data Providers (see main text). Behind each data provider, an information button is given that opens a pop-up-window with further information on the database and the url to the database or information page (Fig. 2 – main text).

All information presented about data providers is retrieved using the core web service function no. 9 (Table 1, Suppl. material 1).

Environmental data

With this function environmental data such as the WFS layer can be uploaded to the AP (> Data - Environmental data - Create layer). The url address can be pasted here directly or the user can choose from given urls by picking one from the list (Fig. S2). At present, WFS layer providers are the SLU GeoServer, the SLW GeoServer, the Swedish Agency for Marine and Water Management, the global data on human impact and the Jordbruksverket´s (the Swedish Board of Agriculture) GeoServer. If a provider is chosen, the service url is given and the user can see a list of available layers (title, namespace, name).

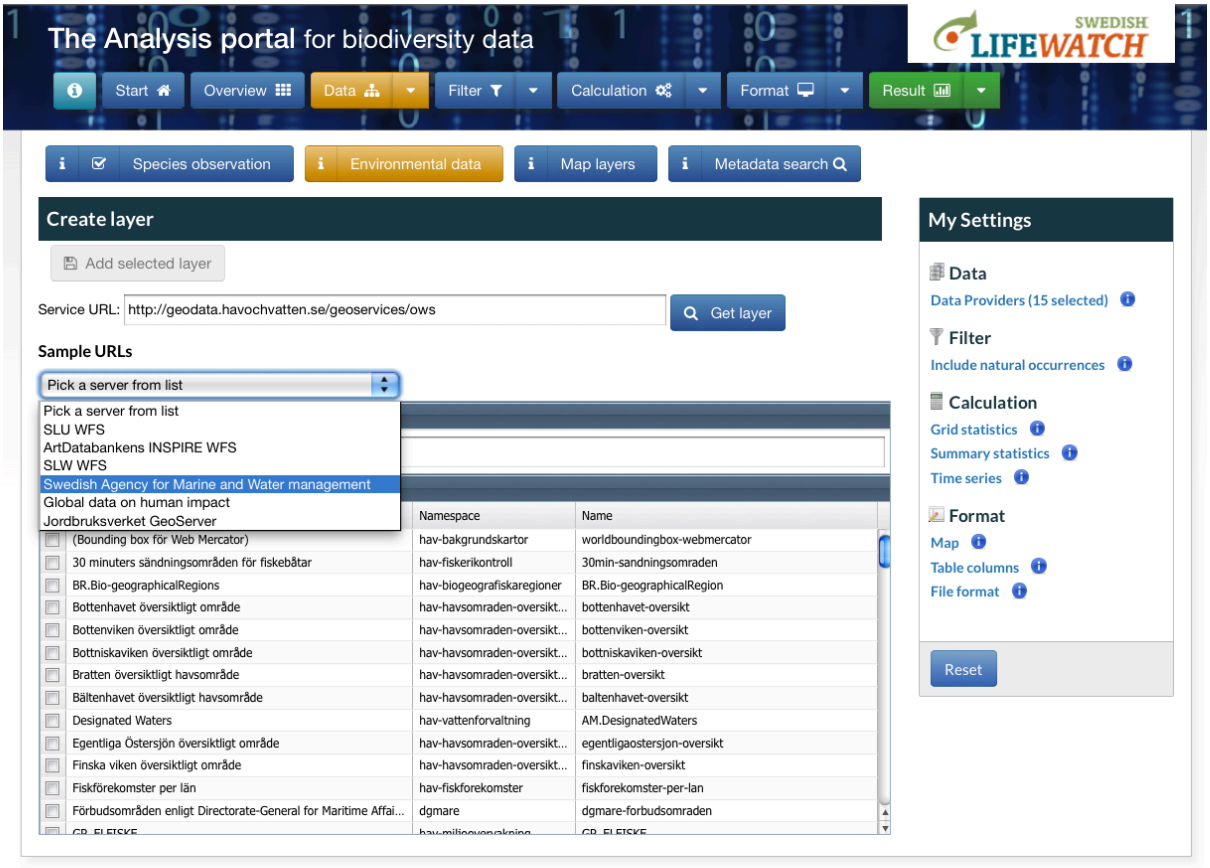

Figure S2: Possibility to add environmental data as WFS layer(s) to the AP.

To retrieve the information from the Web Feature Service (WFS) that is needed for listing layers the standard method GetCapabilities is used. After a data layer has been added to the list of layers it is possible to filter the chosen data in the AP. This functionality is based on the standardized filter syntax that can be used when retrieving data using the standard method GetFeatures. To enable the construction of the interactive filter builder where the user can select possible data fields from a drop down list, the standard function DescribeFeatureType is used.

Map layers

The map layer function is similar to the one for environmental data, where map layers in WMS format can be uploaded or chosen from a provider library (> Data - map layers - add map layer) (Fig. S3). The WMS format allows no calculations on the dataset, in contrast to the WFS-format of the environmental data, but is presented as an overlay in the map result. The main map layer providers are the SLU GeoServer, the SMHI - open data (climate and precipitation map layers), SGU (Geological Survey of Sweden) GeoServer, the Jordbruksverket´s (the Swedish Board of Agriculture) GeoServer (maps on for example water use, grassland, nitrate-sensitive areas), the Swedish Agency for Marine and Water Management´s (marine map layers on for example protection areas), the Baltic Sea Bathymetry database v0.9.3, Metria (INSPIRE geographical information on for example the Swedish NATURA 2000 network, other protected areas based on the EC bird- and habitats directives) and Vic Natur Metria at the Swedish Environmental Protection Agency (terrestrial protected areas like national parks, habitat protection and culture reserves).

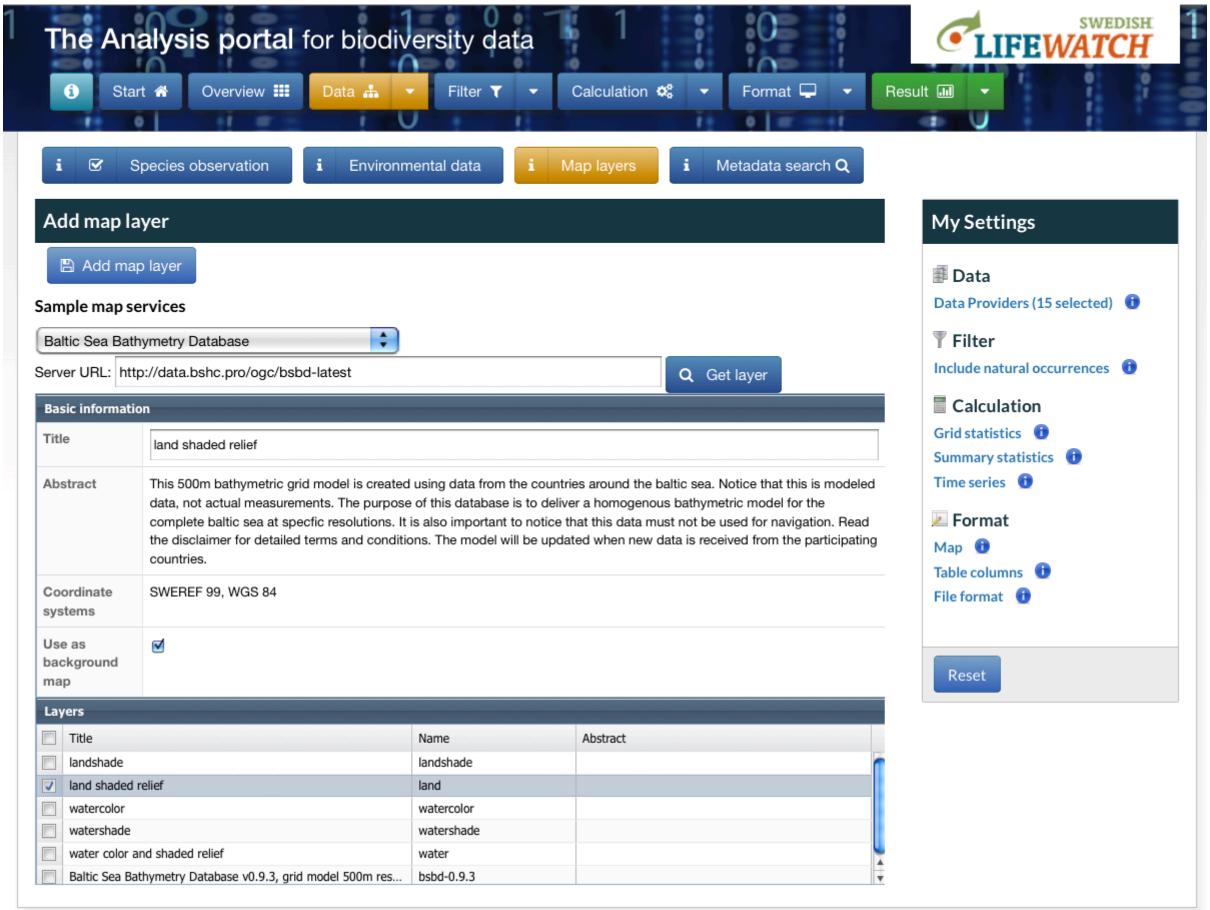

Figure S3: Possibility to add map layer(s) as WMS to the AP. Here the example of land shaded relief from the Baltic Sea Bathymetry Database.

To retrieve the information from the Web Map Service (WMS) that is needed for listing layers, the standard method GetCapabilities is used.

Metadata search

Different metadata portals are listed and linked to this page (> Data - Metadata search), and make it possible to search for more Swedish and international datasets and map and web services. At present, there are five metadata search functions available: *1)* ECDS (Environmental Climate Date Sweden), *2)* Geodata – Sweden bit by bit, *3)* *Biodiversity*Catalogue - The Biodiversity Sciences Web Services Registry, *4)* SMHI – open data catalogue, and *5)* SGU GeoServer (Fig. 5 – main text).

This view currently does not utilize any web services.

2. Filter

Occurrence

The occurrence filters (> Filter - Occurrence) allow settings to be made between presence and absence data (Fig. S4). Presence is divided into natural and unspontaneous occurrences. The latter means occurrence sites that are caused by non-natural distribution. The absence function allows reports to be included of absence in suitable habitat or at sites where previous occurrences were reported. The absence filter is quite useful for ecological niche modelling, where absence data are sometimes required for a certain algorithm. The user has to keep in mind the sample method when using absence data. Two data providers connected to the AP that deliver reliable absence data are NORS and SERS, using the methods of electric fishing and net-fishing (see also Data provider for more information – main text).

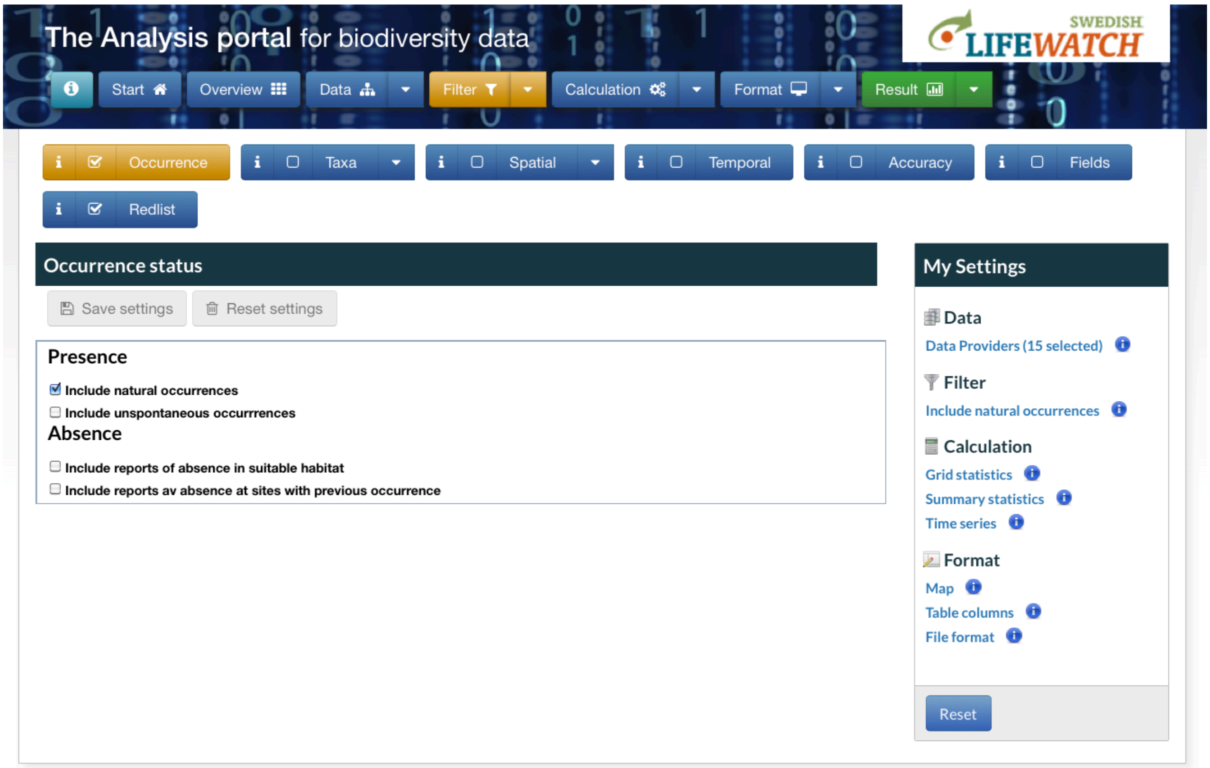

Figure S4: Filtering data with the occurrence status presence/absence functions.

The settings that are handled on this filter view correspond to the species observation search criteria no. 9-12 Table 2 (Suppl. material 2).

Taxa

Taxonomical filtering allows to searches for the taxon name, an input of taxon id list or by searching taxa using selected habitat factors and other taxon attributes. Searching by name (> Filter - Taxa - Search by taxon name) is possible by scientific name or Swedish name of the species. Filtering by genus name also gives a list of all species belonging to the genus, which can then be added to the filter list (Fig. S5).

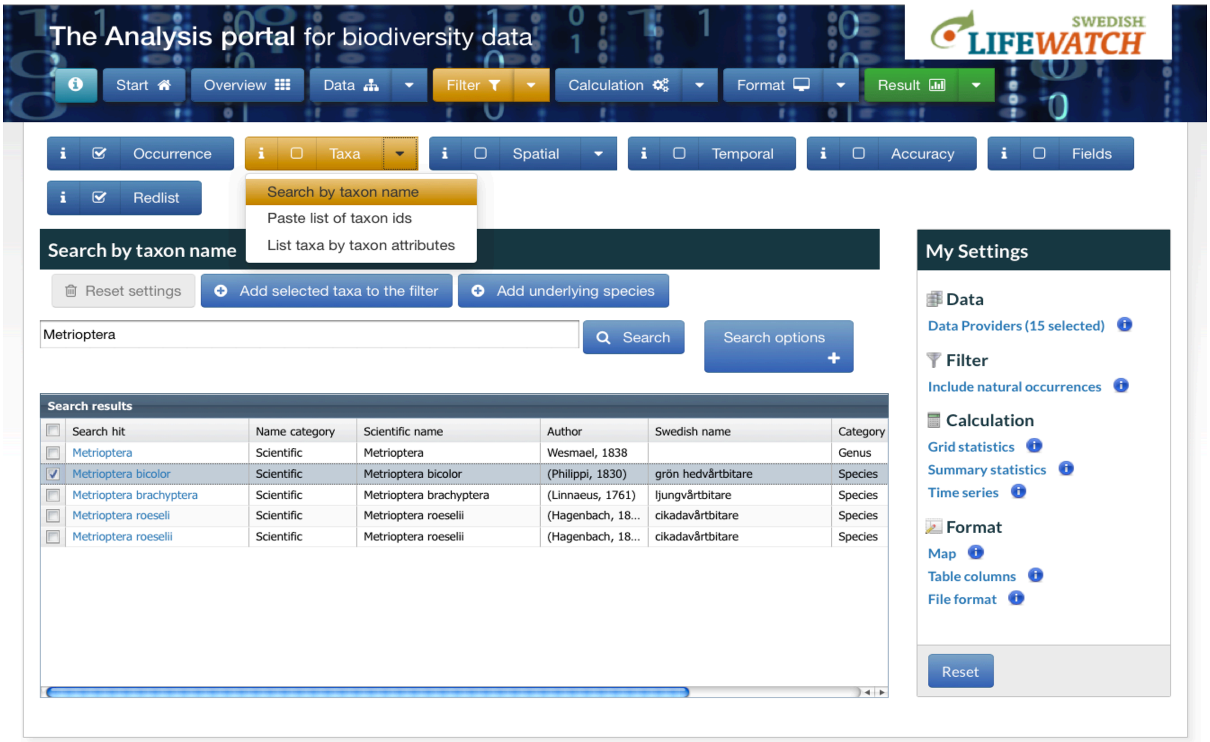

Figure S5: Filtering data with the taxon name function, here for the two-coloured bush-cricket *Metrioptera bicolor*. It is possible to choose all underlying species in a family or order.

Searching by taxon ids (> Filter - Taxa - Paste list of taxon ids) allows specific searches of protected species (e.g. red-listed species) or character species used in monitoring and inventory programs. Consulting companies or county/municipality administrations commonly uses this function. The AP offers a clipboard to paste the taxon ids, but also gives a link to search for the taxon id with the help of Dyntaxa (Fig. S6).

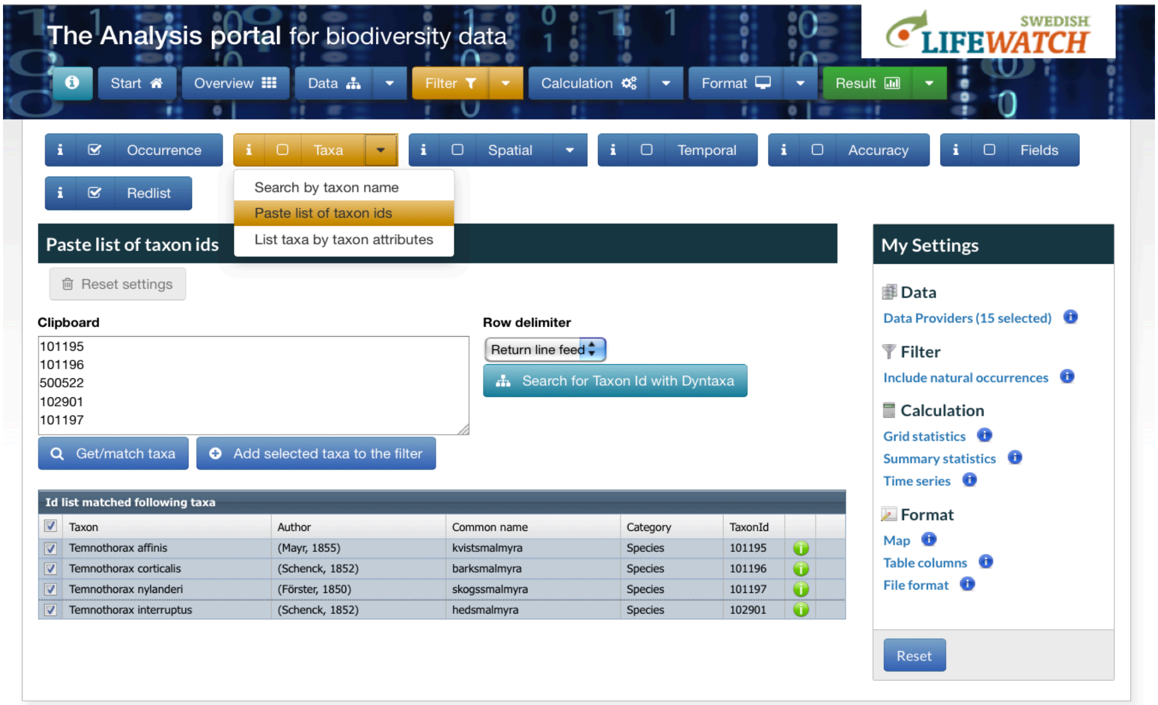

Figure S6: Filtering data with the help of a taxon id list, here for some ant species.

The third filter is for searching by taxon attributes (> Filter - Taxa - List taxa by attributes), which means habitat-related factors like habitats defined according to the EU habitat directives (e.g. moss, forest, lakes, open coast, marine areas), substrate (e.g. air, water, other organisms, man-made structures), impacts (e.g. climate impacts, land-use, hunting, water regulations, aqua culturing, exploitation) and other species traits (e.g. ecological group, dispersal, generation time) (Fig. S7).

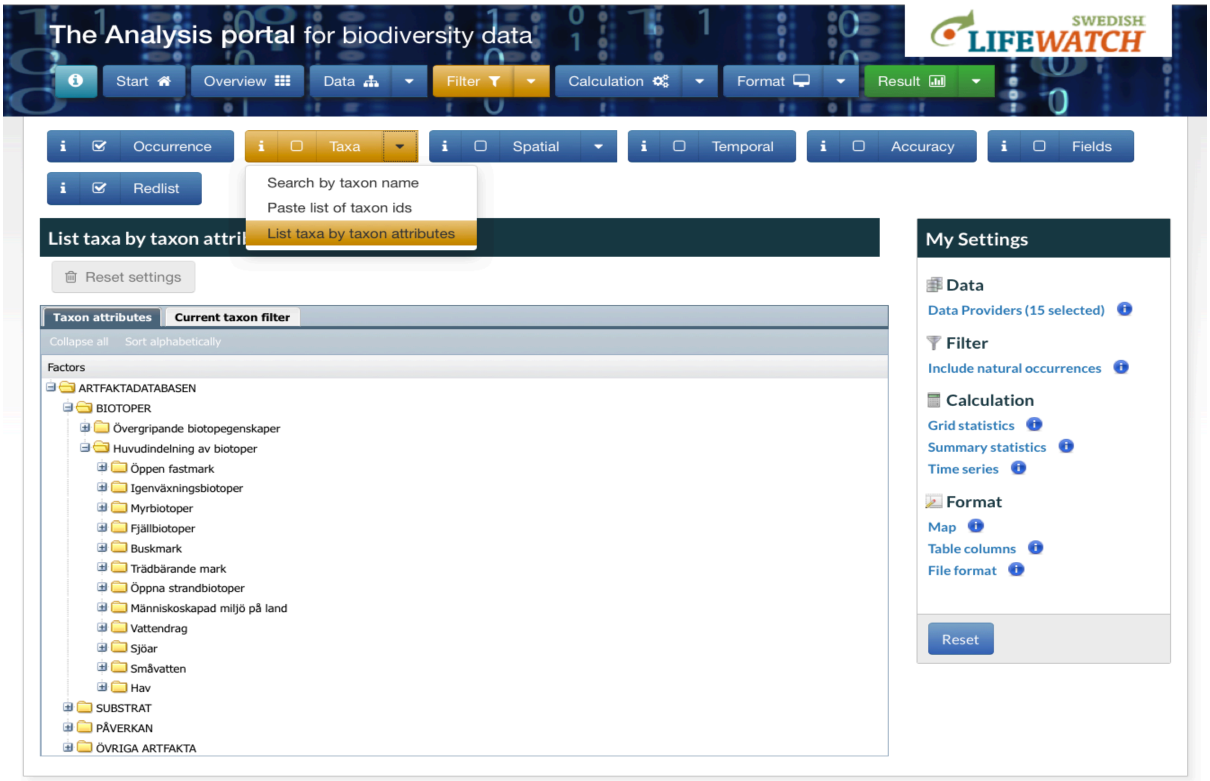

Figure S7: Filtering data by taxon attributes.

All the taxon filter views affect the same property of the species observation search criteria (Table 2), namely the list of TaxonIds (no. 1). When this list is set directly from a list of Dyntaxa taxon ids provided by the user, the corresponding taxa with recommended name is shown in a list. This list is obtained from the taxon service using method no. 3 (Table 1). When the user instead choose to set the taxon filter by search for taxa one at a time based on some taxon name string, the core web service method 2 is used (Table 1). Once a taxon is found and selected, the user may either add the taxon to the filter list of taxa or choose to retrieve all underlying species. The latter procedure utilizes method number 3 (Table 1). If the user happens to search for taxa that have been lumped and consequently are no longer valid, it is possible to utilize method 4 (Table 1) in order to retrieve information about which taxon should be used instead.

As alternative to the name based retrieval of taxa to add on to the species observation search criteria, it is possible to use method 5 (Table 1), which utilizes taxon attributes to list taxa. To retrieve the factors that can be selected for taxon listing, method 6 (Table 1) is used. Method 5 is also used for retrieving taxa based on Red List Categories as Red List Category is one of the factors handled by the Taxon Attribute Service.

Spatial

The spatial filter (> Filter – Spatial) is divided into four different functions. It is possible to draw a geometric figure (e.g. rectangle, polygon) and move and edit it or select a certain number of different ones on the map (> Filter – Spatial – Draw Geometry) (Fig. S8).

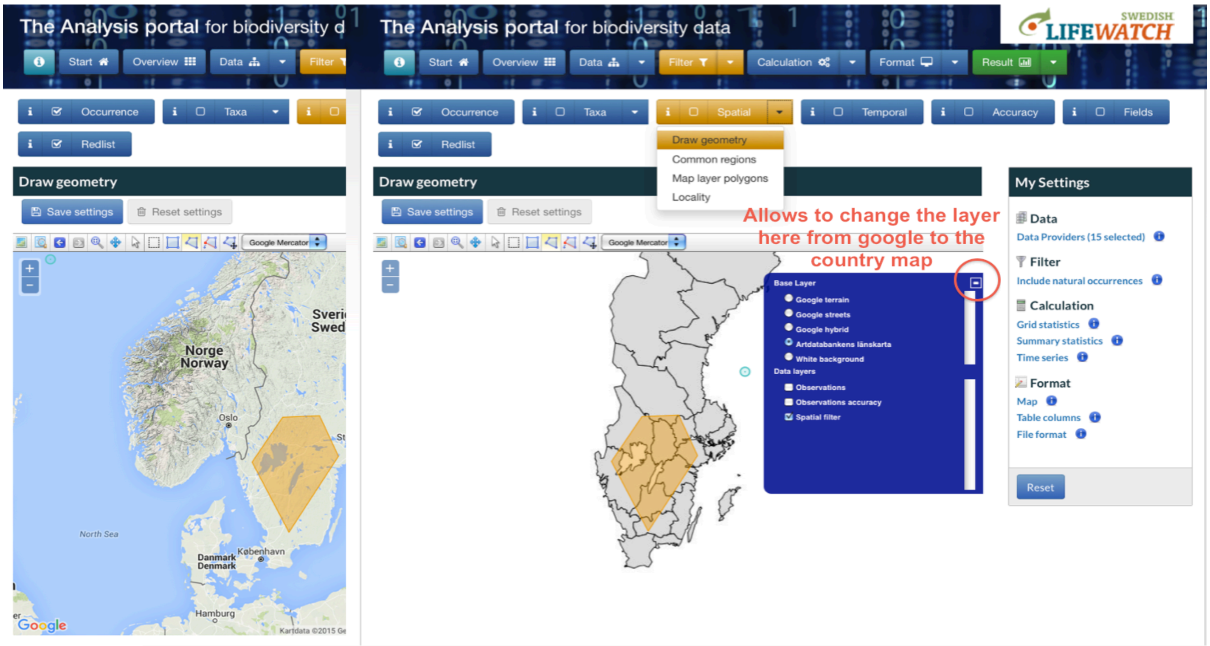

Figure S8: The filter function to draw a polygon. Here the Google map was changed as base layer to the county map of the Swedish Species Information Centre.

Map layer polygons can also be uploaded as spatial filters in GeoJSON file format (encoding geographic content in JavaScript Object Notation) (> Filter – Spatial – Map Layer Polygons) (Fig. S9). For example, the user can choose a taxon (> Filter - Taxa - Search by taxon name) and download the species observation or species richness grid maps (see under Result for more information) and save the grid map as a GeoJSON file on the computer. Under Filter – Spatial – Map Layer Polygons the user can then upload the GeoJSON file again and use it as a filter for further analysis.

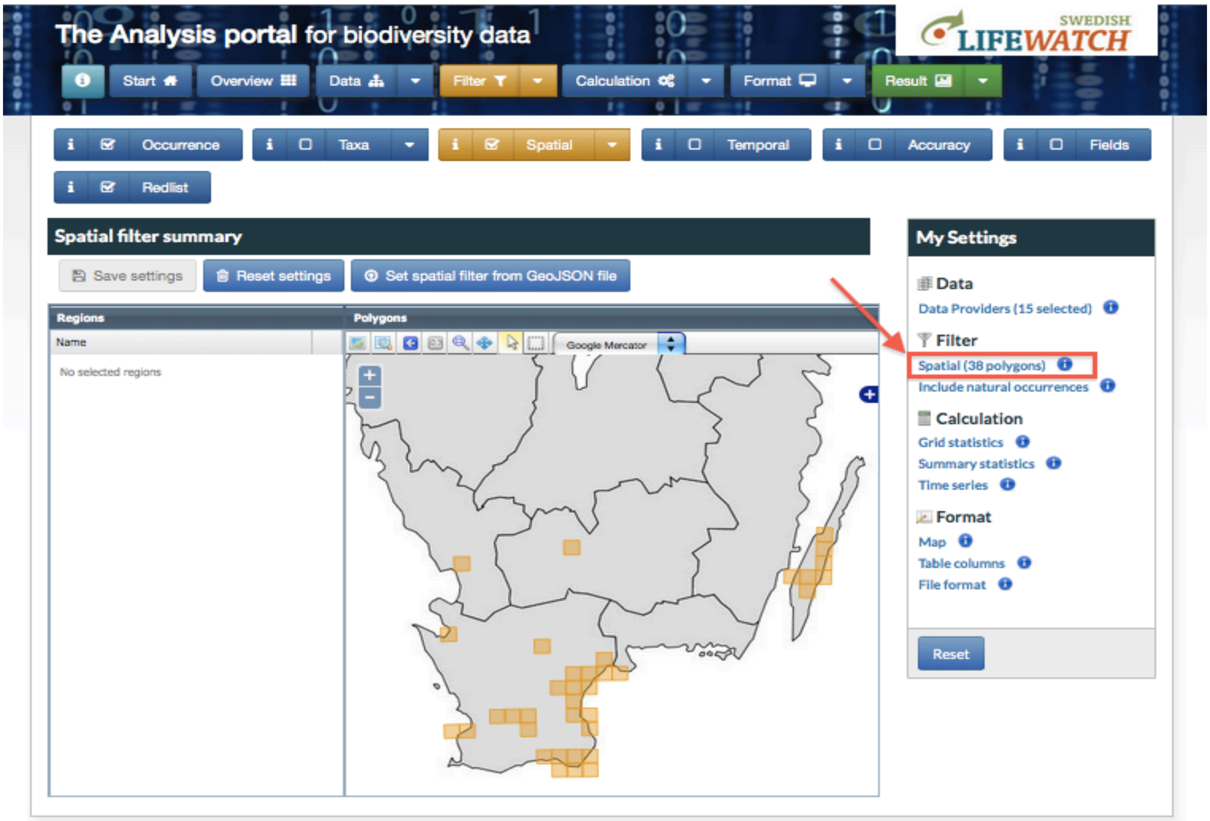

Filter S9: Using the map layer polygons as spatial filter. Here a GeoJSON file was uploaded and used as filter.

The AP also offers the spatial filter on common Swedish regions through a list of provinces, counties and municipalities that the user can choose from (> Filter – Spatial – Common Regions) (Fig. S10). It is also possible to search by locality name (> Filter – Spatial – Locality) (Fig. S11).

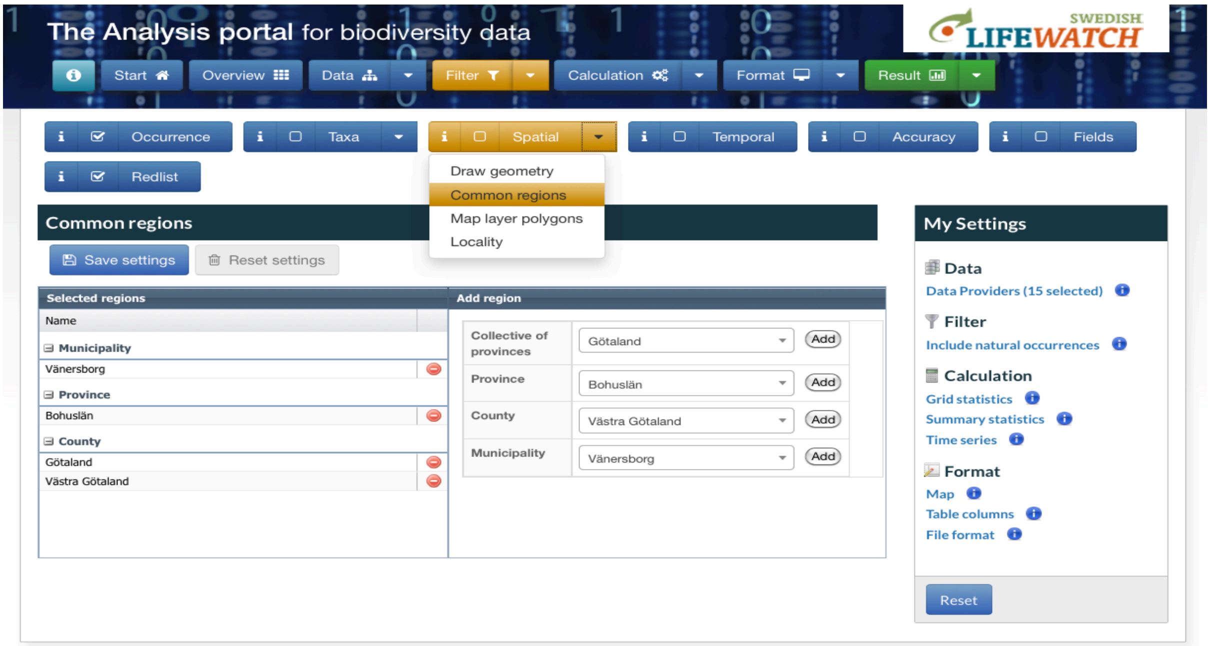

Figure S10: Filtering function by common Swedish regions.

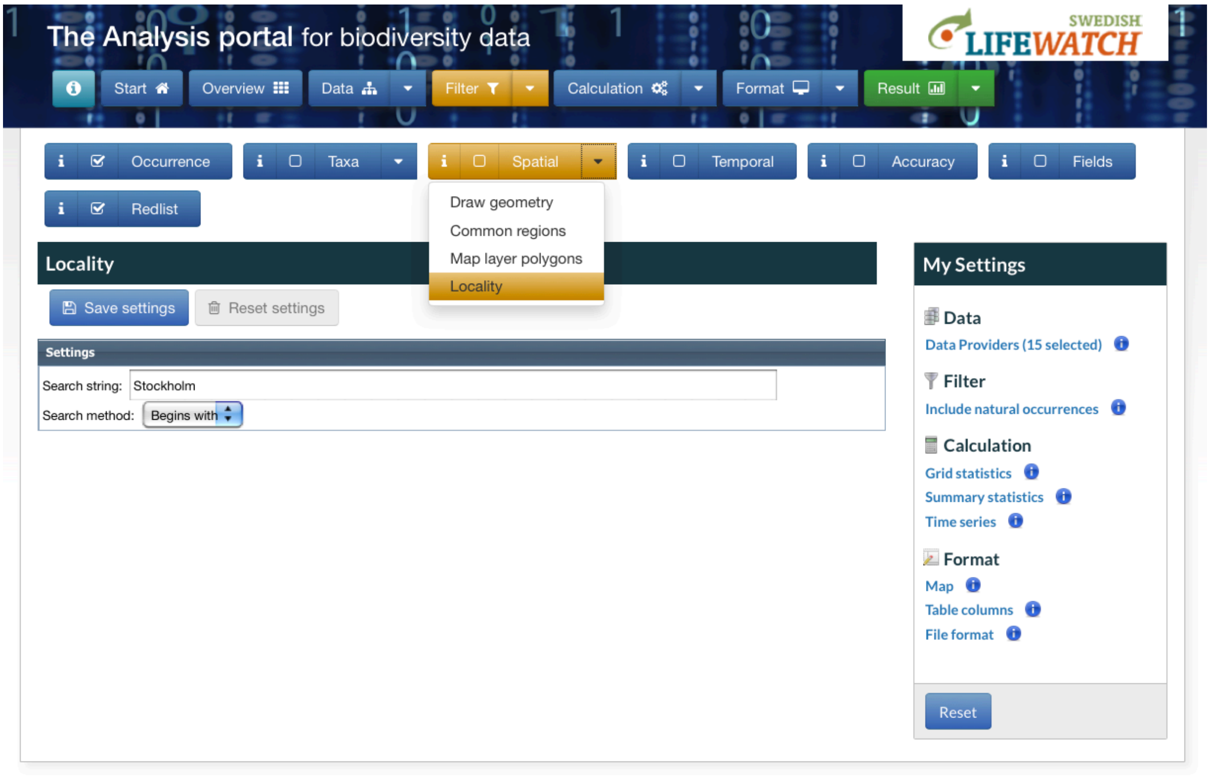

Figure S11: Spatial filtering by locality name.

Technically, when it comes to setting the species observation search criteria based on various polygons drawn or selected by the user, criterion no. 16 (Table 2), which can take a list of search polygons is used. When selecting a list of named regions, like municipalities or counties, criterion no. 17 can be used. This requires the region GUIDs to be known. In order to fill the dropdown lists with region names, the core web service function no. 7 (Table 1) is then used. With this method it is possible to retrieve all the region names and GUIDs based on a specified region category.

Temporal

The time-based search function (> Filter – Temporal) is given by entering the observation, registration or change date. By default this function is ignored, but can be changed to the exact date or annual approach (Fig. S12).

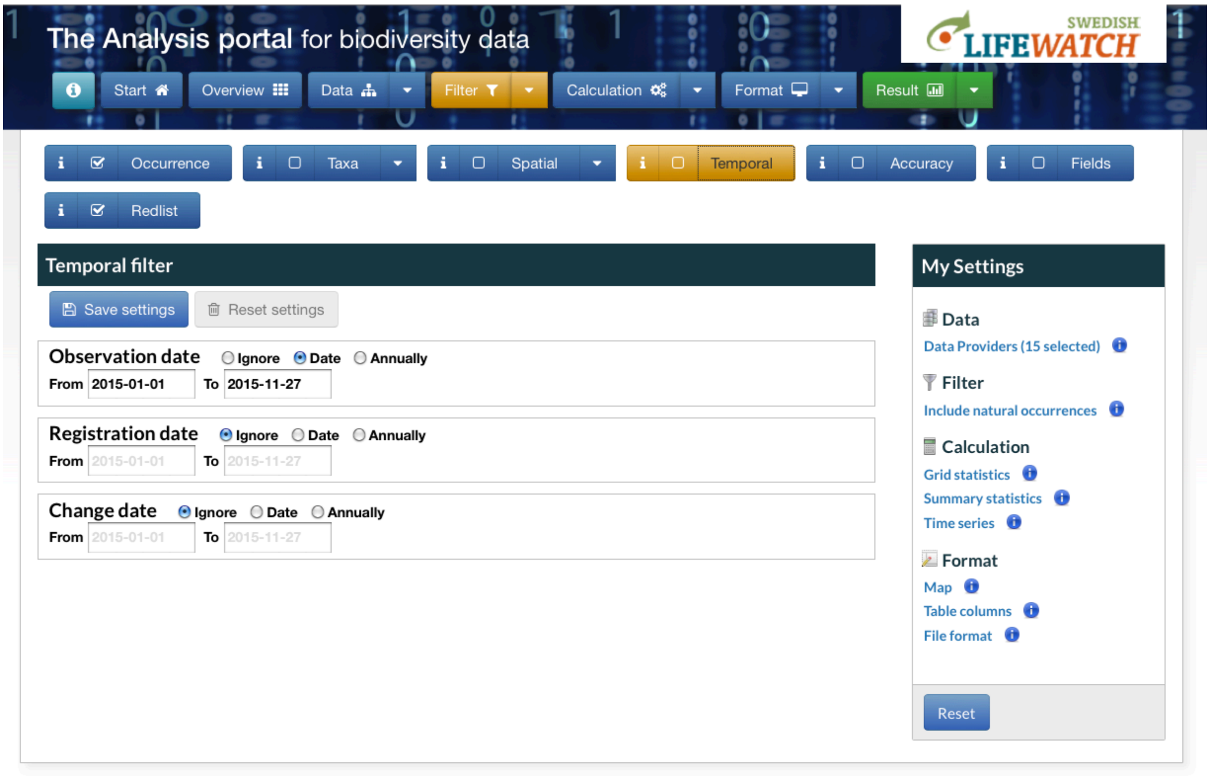

Figure S12: Temporal filter in the AP. Here the chosen observation date first of January until 27th of November 2015.

The settings that are handled on this filter view correspond to the species observation search criteria nos. 22 and 26-27 (Table 2).

Accuracy

This filter section (> Filter – Accuracy) is intended to handle different types of uncertainty which may otherwise have a negative impact on the result. When dealing with species observations, three main uncertainties may be relevant to consider when selecting data suitable for a particular analysis, i.e. spatial, temporal and taxonomic uncertainty. The Species Observation Search Criteria used in the Web Service methods support all these dimensions of data accuracy (Table 2). However, only spatial accuracy can be set as filter in the AP interface (Fig. S13). Spatial accuracy is handled by criteria properties nos. 19-21 (Table 2). Temporal accuracy is handled for each temporal search criterion (nos. 22, 26-27) separately. Taxonomic uncertainty is evaluated by certain expert validators and is expressed in terms of a number of validation statuses (criterion no. 2, Table 2).

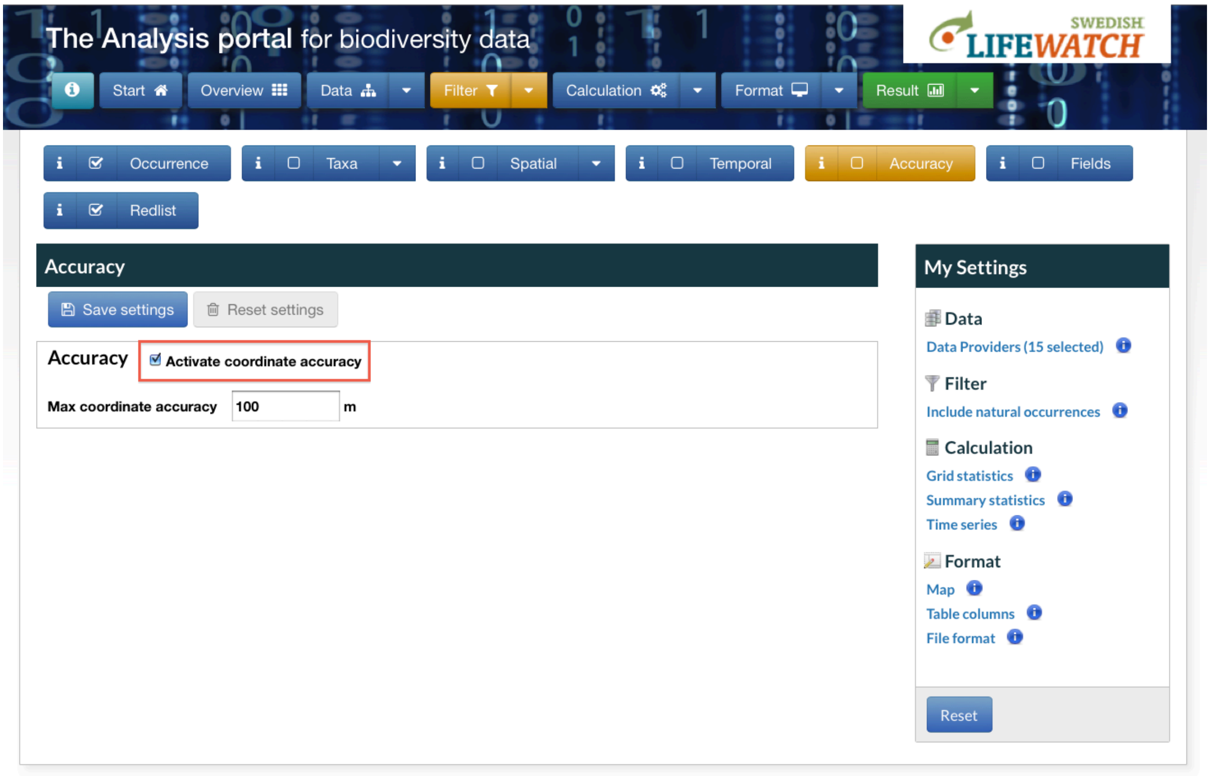

Figure S13: The AP offers the possibility to activate coordinate accuracy.

By default the spatial accuracy filter is not activated, which means that all species observation records will be retrieved regardless of the value in the Darwin Core field coordinateUnceratintyInMetres. However, the user may set a maximum accepted value if needed.

Spatial accuracy of species observations is of great importance in many types of analysis. For example, when working with ecological niche modelling there is a need to find relationships between different environmental factors and the occurrences of the focal species. If the spatial resolution of the occurrences is too poor in relation to the spatial resolution of the environmental information, then serious errors are very likely to appear. Similarly, when calculating species richness per grid cells it is an advantage to exclude observations with less spatial accuracy than the size of the grid cells.

When analysing species observations it is often and advantage to use only data where the spatial, temporal and/or taxonomic precision is more accurate than a certain threshold value. In the Species Observation Search Criteria, spatial accuracy is specified as the maximum radius measured in metres from the reported coordinate within which the observation was made. All observations handled by Swedish LifeWatch have a measure of this uncertainty. The uncertainty circle may include both the expected error in the coordinate estimation for the actual observation and/or the area of the habitat patch within which the observed individuals are expected to move around.

It is possible to filter species observations based on detailed content of specific data fields (> Filter – Fields). Depending on the data type of the data field, different operators can be applied. 'Equal' and 'Not equal' can be applied on all searchable fields. For fields containing

text, which is the case with most Darwin Core terms, it is also possible to apply one of the following operators: ‘Begins with’, ‘Contains’, ‘Ends with’ and ‘Like’. For numerical fields it is possible to use ‘Greater than’, ‘Greater than or equal to’, ‘Less than’ or ‘Less than or equal to’.

In the view where the field-based filter for species observations can be specified (Fig. S14) it is possible to list several combinations of field name, value and operator which can be treated either as an ‘and’ or an ‘or’ statement. When using the ‘and’ button, the added combinations of field, value and operator have to be true regarding the data record in order to fit the species observation search criteria. When using the ‘or’ button only one of the specified combinations has to be true.

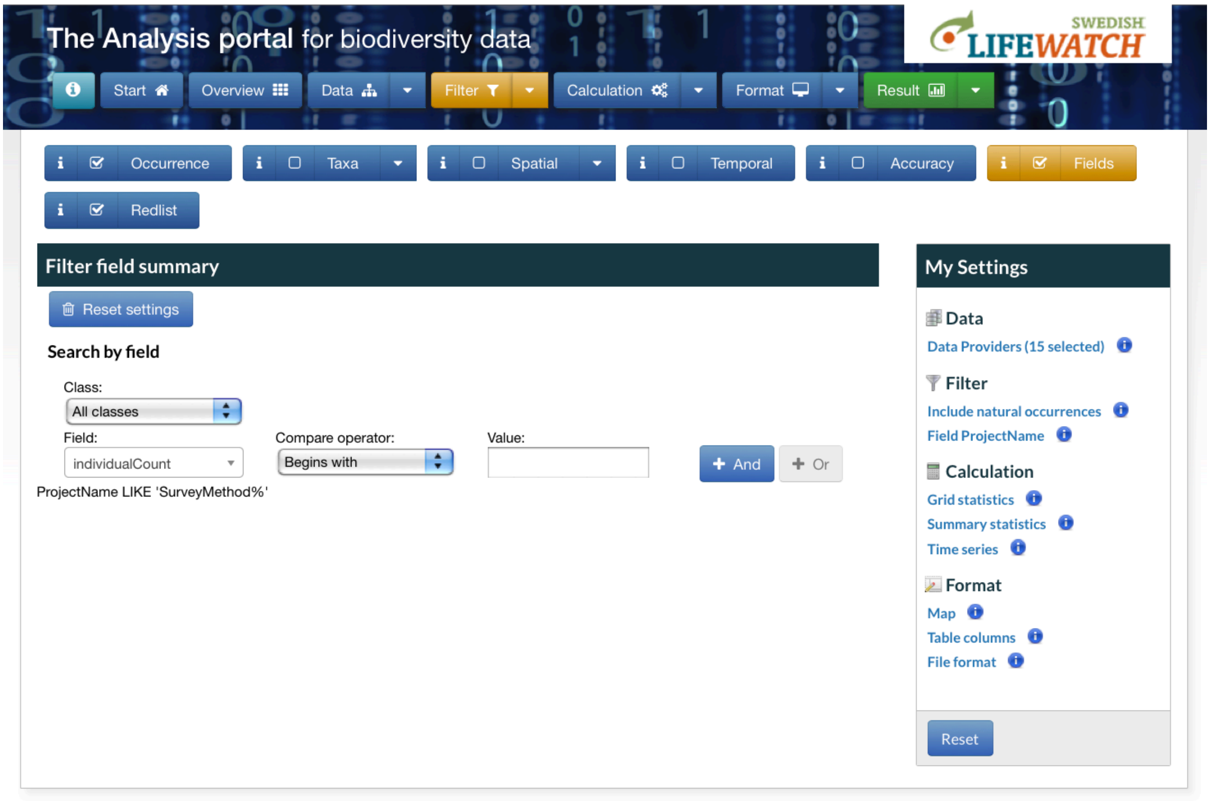

Figure S14: The AP offers the possibility to activate coordinate accuracy.

With this filter it is possible to select only observations where for example individualCount is greater than 100 or where the field habitat contains the word ‘äng’, which is the Swedish word for ‘meadow’.

Selected combinations of fields, operators and searched values are set using a specific field search criterion (no. 13, Table 2), which is a part of the SpeciesObservationSearchCriteria object used when retrieving observations or observation statistics from the core web services.

Red List

This function (> Filter - Red List) makes it possible to search by Red Listed taxa in the AP (Fig. S15). The user can choose between the different red list categories following the international specifications of the IUCN (IUCN 2001) or choose them all together. This filter is useful in combination with a spatial filter.

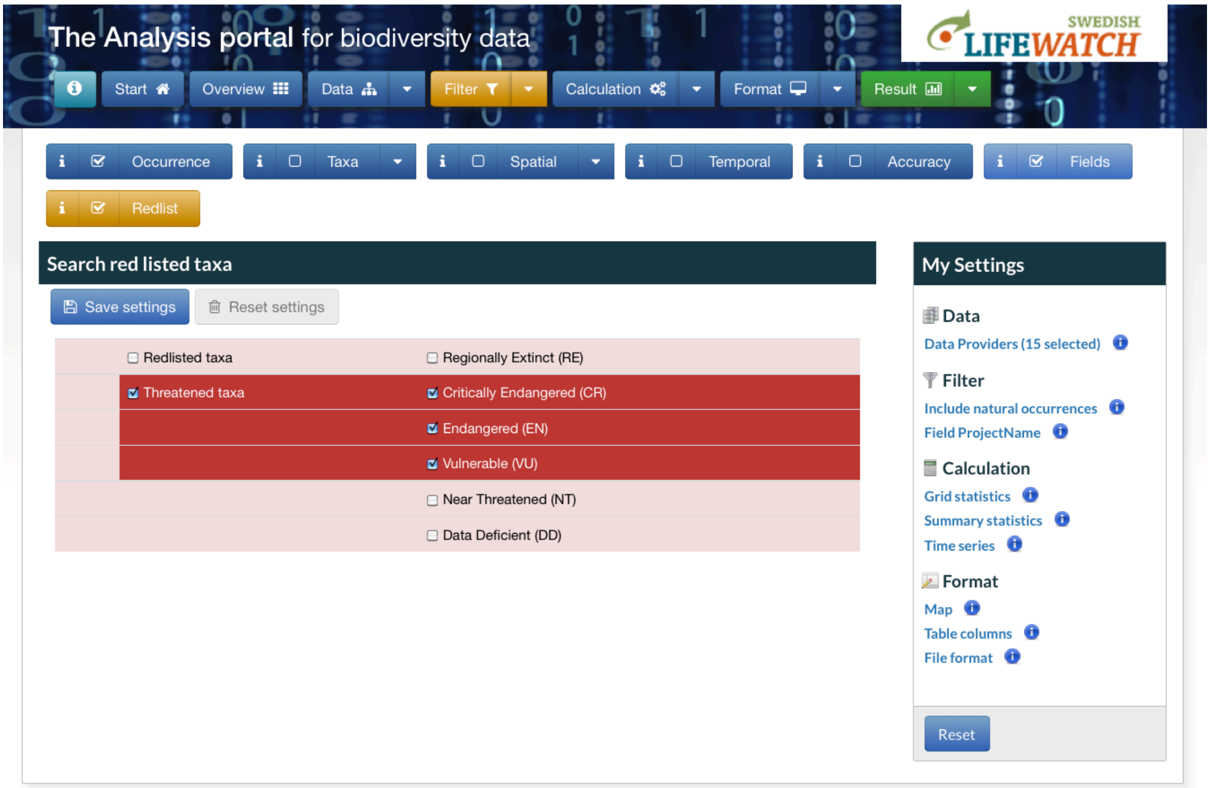

Figure S15: Filter function to search after red listed taxa following the IUCN criteria.

The settings that are handled on this filter view correspond to the species observation search criterion no. 3 (Table 2).

3. Calculation (data processing)

Summary statistics

Different types of summary statistics (> Calculation - Summary statistics) can be calculated from the selected observations and/or map layers. The user can choose between calculations from the number of observations and/or the number of species (Fig. S16). If the user is running an analysis that includes environmental data (WFS layer), there is the possibility to select a certain layer and obtain summary statistics per polygon and a report on the summary statistics. Both can be downloaded as Excel files (see also 'Result' for more information).

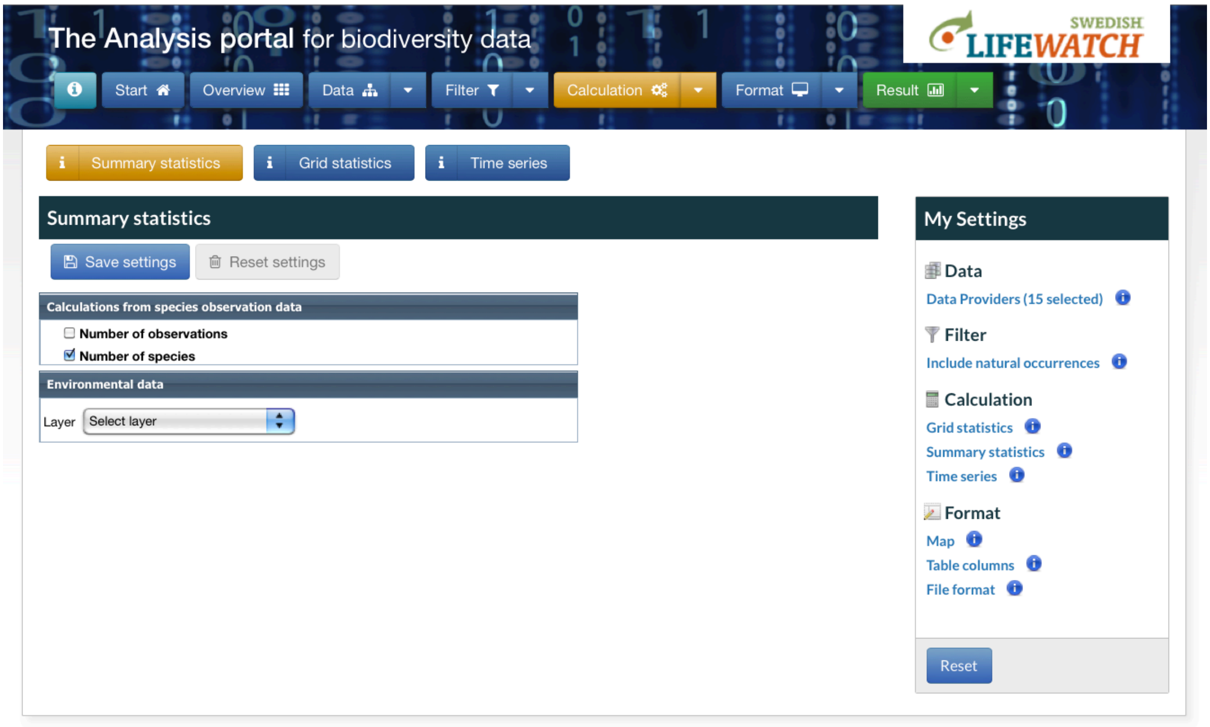

Figure S16: The AP offers the possibility to change settings for the summary statistics.

Today, the usefulness of this view is rather limited as only two types of summary statistics can be selected from. However, many more indexes and estimates are expected to be handled in the AP in future. As some summary statistics may take time to calculate this view can give the user the opportunity to exclude statistics, which are of no interest and thereby speed up response times for various result views and downloads.

The selection of polygon data layer affects which layer should be used when calculating summary statistics using the download method Summary statistics per polygon (see also > Result – Tables – Summary Statistics Per Polygon).

The settings provided by the user from this view affect the usage of core web service functions 17-18 (Table 1).

Grid statistics

Grid statistics can be changed by the grid parameters (>Calculation – Grid statistics), namely the grid size in metres and the coordinate system (SWEREF 99, ESPG:3006 or RT 90, ESPG:3021). The statistics can also be changed to generate all grid cells, even empty ones. The calculations for the grid statistics are by default the number of observations and taxa. Similar to the summary statistics, if the user is running an analysis that includes environmental data, the layer can be selected and calculated by count, area or length (Fig. S17).

This view sets essential input parameters for the core web service methods that calculate grid-based summary statistics, i.e. nos. 22, 23, 25 and 26 (Table 1), which are used in a number of results and download functions in the AP.

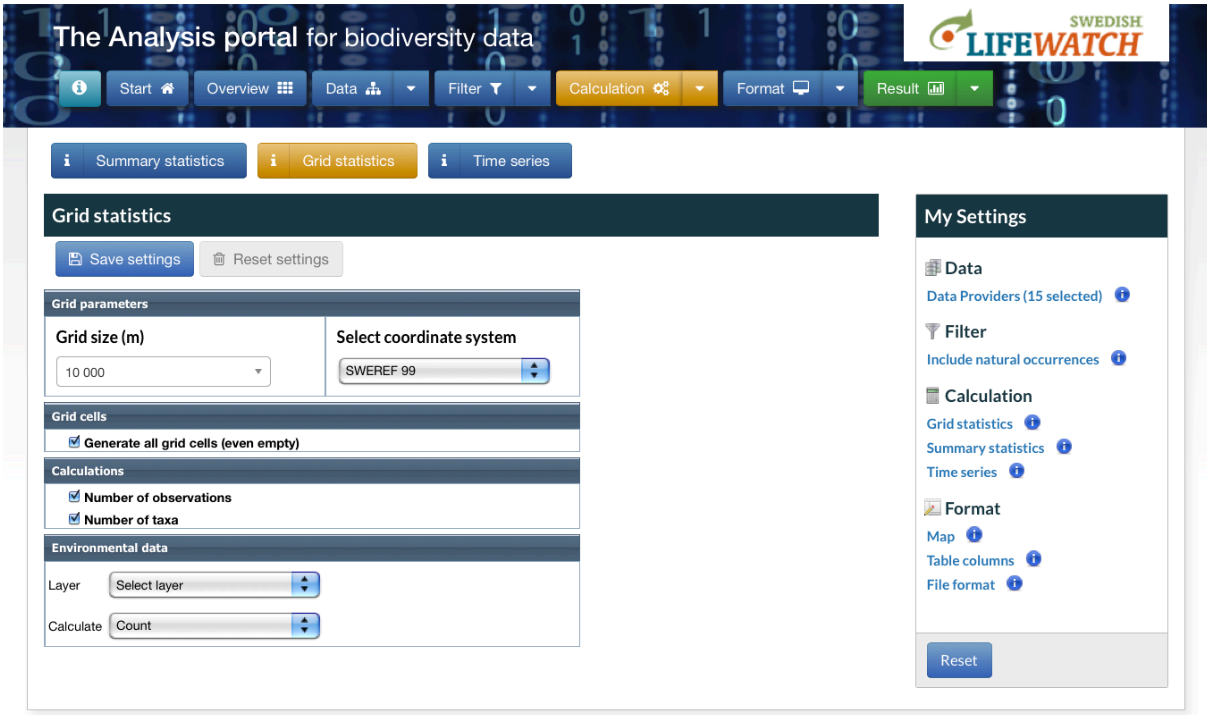

Figure S17: The grid statistics can be changed in the AP.

Time Series

The user can change the setting for the time series (> Calculation – Time series). Time series can be set on the periodicity of yearly, monthly, weekly, daily, month of the year, week of the year and day of the year (Fig. S18).

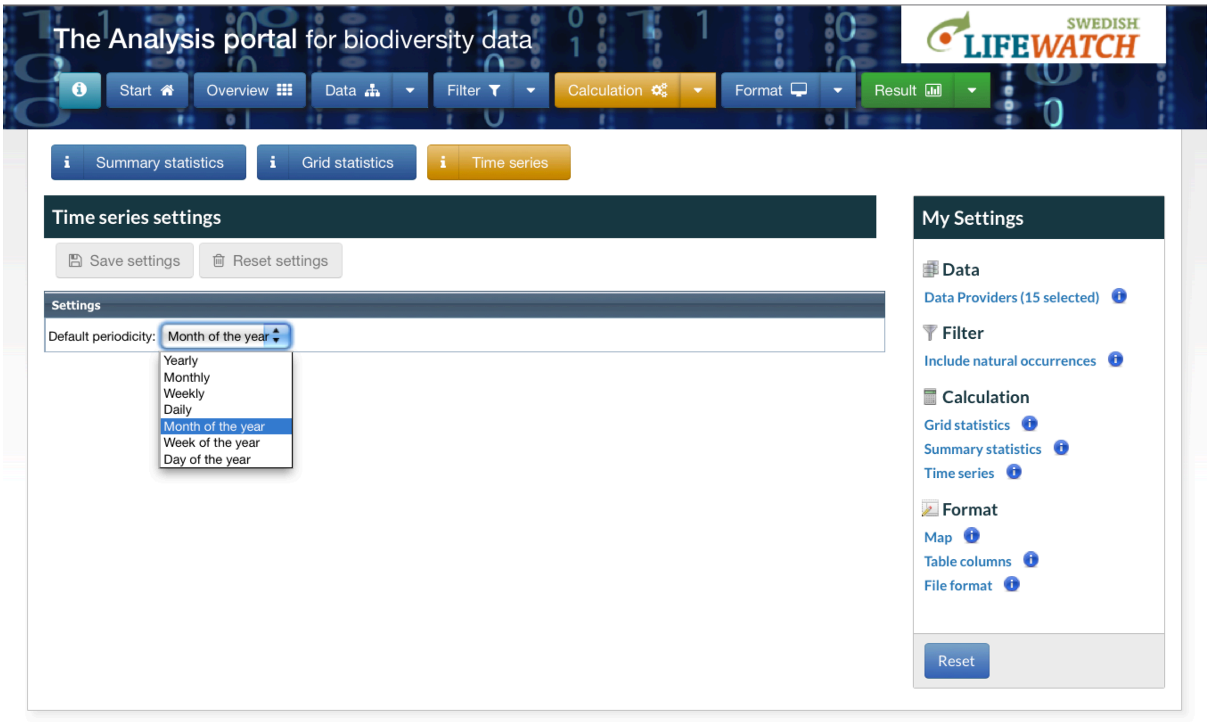

Figure S18: Analysis on time series allow different settings on periodicity.

This view sets essential input parameters for the core web service method that retrieves time step specific counts of observations (no. 21, Table 1).

4. Format (presentation and export settings)

Map

To present the available data in the AP, the user can choose the coordinate system under map settings (> Format – Map). Available coordinate systems are Google Web Mercator (EPSG:3857), SWEREF 99 (EPSG:3006), RT 90 (EPSG:3021) and WGS 84 (EPSG:4326) (Fig. S19).

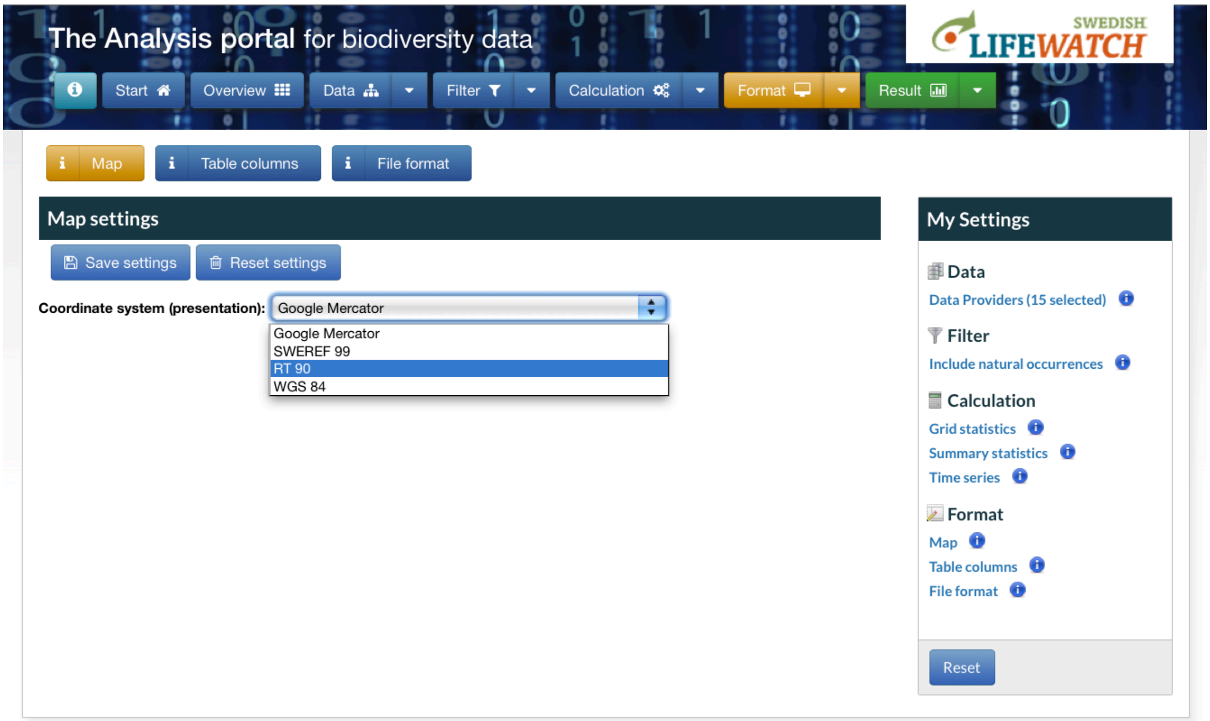

Figure S19: The coordinate system can be changed.

Most core web service methods that handle spatial coordinates either for representations of species observation location in the output or in terms of polygons specified as species observations search criteria require that the coordinate system is specified. This applies for the majority of functions provided by Swedish Species Observation Service and Analysis Service (Table 1).

Table columns

The user can choose which table type the results will be presented in (> Format – Table columns). There are different numbers of columns in the pre-selected column sets, called minimum (16 columns), basic (28), average (49), extended (100) and all (120). The labels can be changed to standard labels (Scientific name, Common name etc.) or Darwin Core labels (scientificName, vernacularName etc.). Behind each column the user can find an information button (Fig. S20), where for example the id number, guid, label, class, type, importance, definition, definition url and remarks are given.

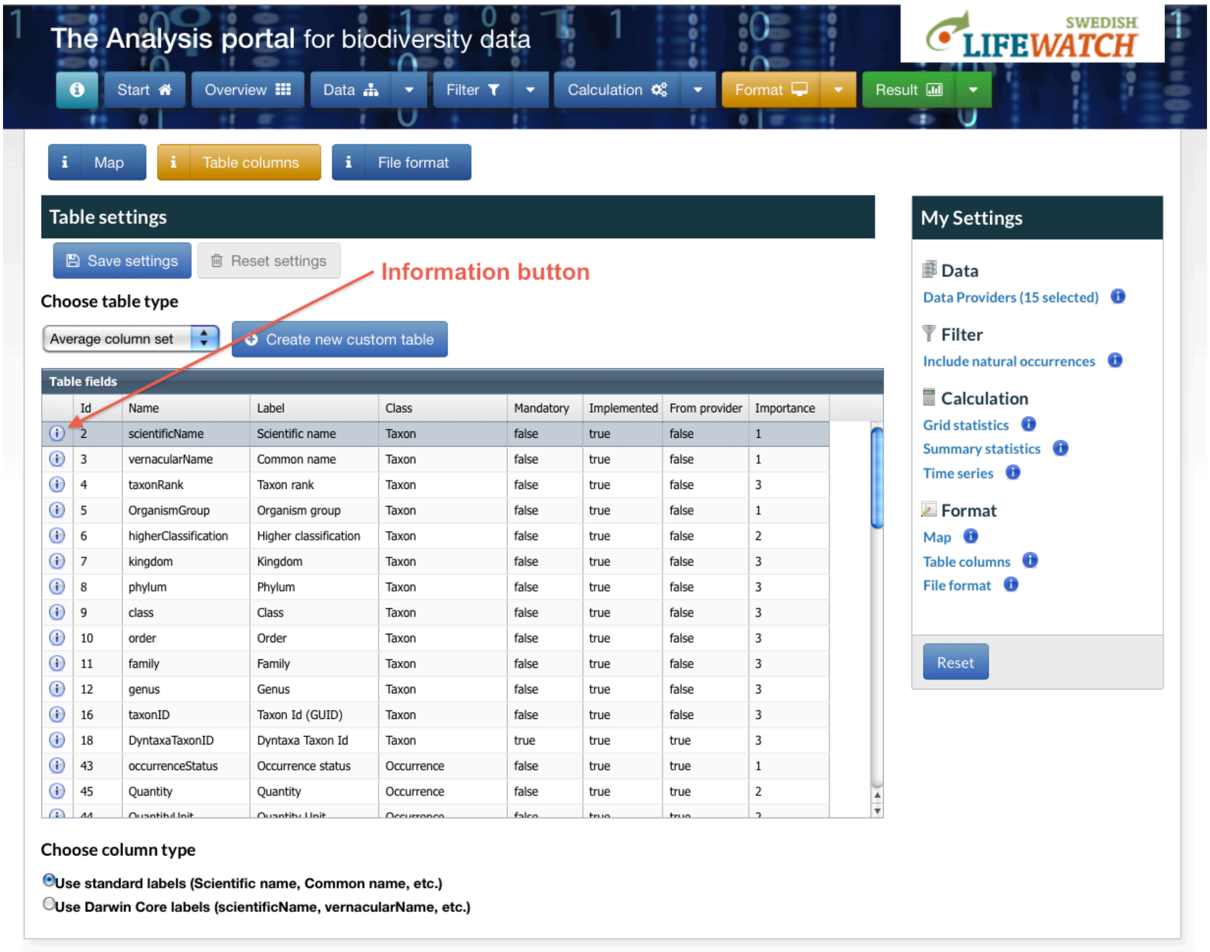

Figure S20: The table type settings allow different column sets and labels.

In this view it is also possible to construct user-defined selections of data columns. If the user is logged in, the column selections can be saved.

All metadata on available data fields that can be selected as columns for different output representations of observation records with the AP are obtained by using a single core web services method (no. 10, Table 1).

File format

Different file format settings exist for downloading the result of the analysis (> Format – File Format). The csv column can be comma, semicolon, pipe or tab separated. The Excel file format is either .xlsx or .xml (Fig. S21).

The different formats that can be selected for downloads in the AP do not affect the use of the core web services as these are based on a specific standardized protocol, i.e. SOAP.

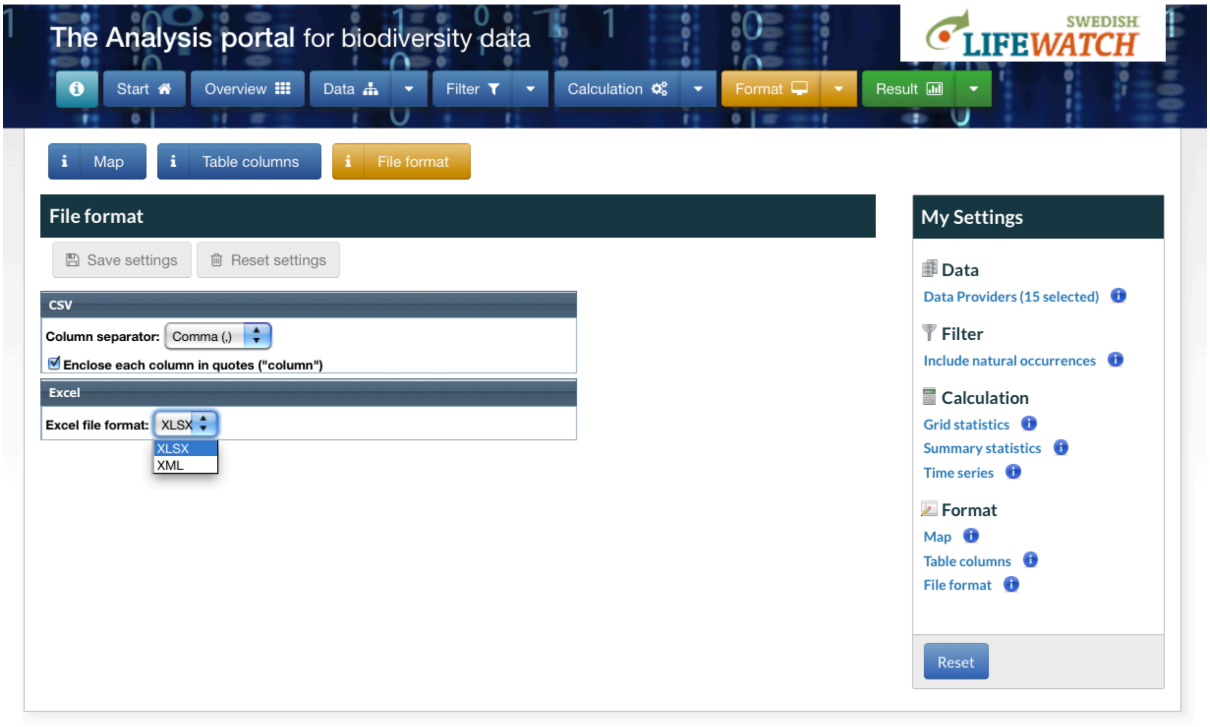

Figure S21: The file format can be changed in the AP.

5. Result

Maps

For showing results, the user can choose the type of map (> Result – Maps). All maps have a toolbar at the top, where different functions are available, e.g. it is possible to change the coordinate system (Google Web Mercator (EPSG:3857), SWEREF 99 (EPSG:3006), RT 90 (EPSG:3021), WGS 84 (EPSG:4326)) or choose between different map layers (base and data layers).

In total, five different map types are available in the current version of AP.

1) The species observation map (> Result – Maps – Species Observation Map), that shows the recorded distribution of the chosen taxa as points (Fig. S22). Clicking on each point gives the information available on the registered data (data provider, data owner, coordinates, date etc.). The observation details can be changed from basic, average, extended or all field sets. The map can be shown in full screen mode, which may improve map navigation and the overall user experience.

This map visualization of species observation utilizes a paged version of data retrieval (function 16, Table 1). With this function it is possible to specify which data field should determine the sort order of retrieved records. In this case observation date are used in a way (descending) that makes this map view particularly well-suited for exploration of the latest observations reported to the connected data providers in the SLW infrastructure.

Paging forward with the navigation button below the map retrieves successively older observations.

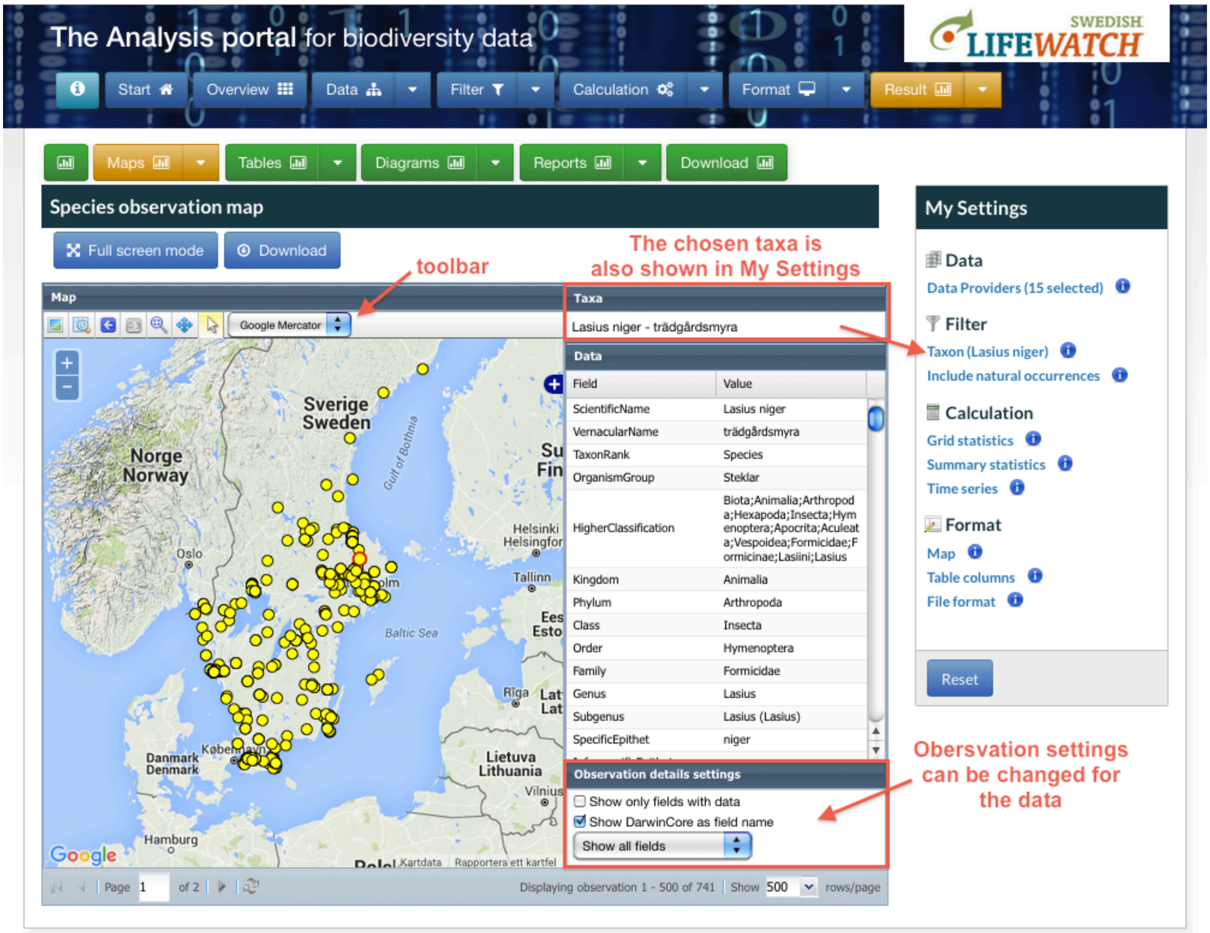

Figure S22: The species observation map for the ant species *Lasius niger.* For the chosen record the information is given in the right-hand panel.

As very few data fields are necessary when just plotting the spatial coordinates as points and the spatial accuracy as circles with the radius set to the DarwinCore term coordinateUncertaintyInMeters, only a few data fields are retrieved initially. First, when the user clicks on a particular point the full list of data fields for that particular record is retrieved from the core web service using method no. 14 (Table 1), which takes record ids as input.

2) The species observation grid map (> Result – Maps – Species Observation Map) presents the number of observation counts per grid. The results can be shown in full screen mode and downloaded as an Excel or GeoJSON file. The user can set the legend by adding a certain number of bins, changing the colour and changing the mode. For each grid cell the borders can be drawn, to make them more visible and the transparency can be chosen in 10% steps. By clicking on a grid cell, the data information is shown in the right-hand panel under data (here field: ObservationCount and Value: 2) (Fig. S23).

The species observation grid map is rendered based on the grid statistics obtained from core web service method no. 22 (Table 1).

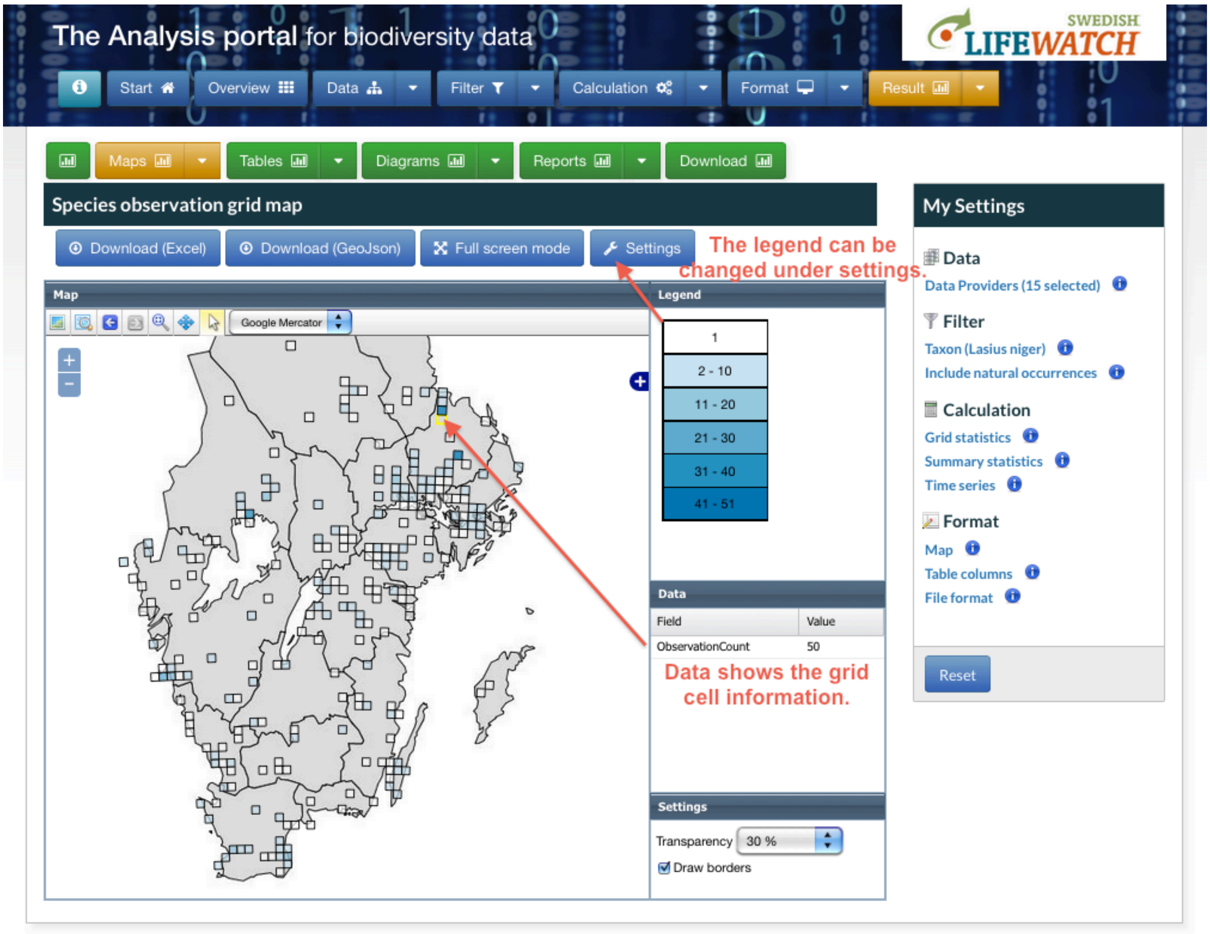

Figure S23: The species observation grid map presents the observation counts per grid for the species *Lasius niger*. The legend can be changed individually.

3) The species richness grid map (> Result – Maps – Species Richness Grid Map) is similar to the species observation grid map, but in addition to the observation count the user also gets the species count for each grid cell (Fig. S24).

This grid map is rendered based on the grid statistics obtained from core web service method no. 23 (Table 1).

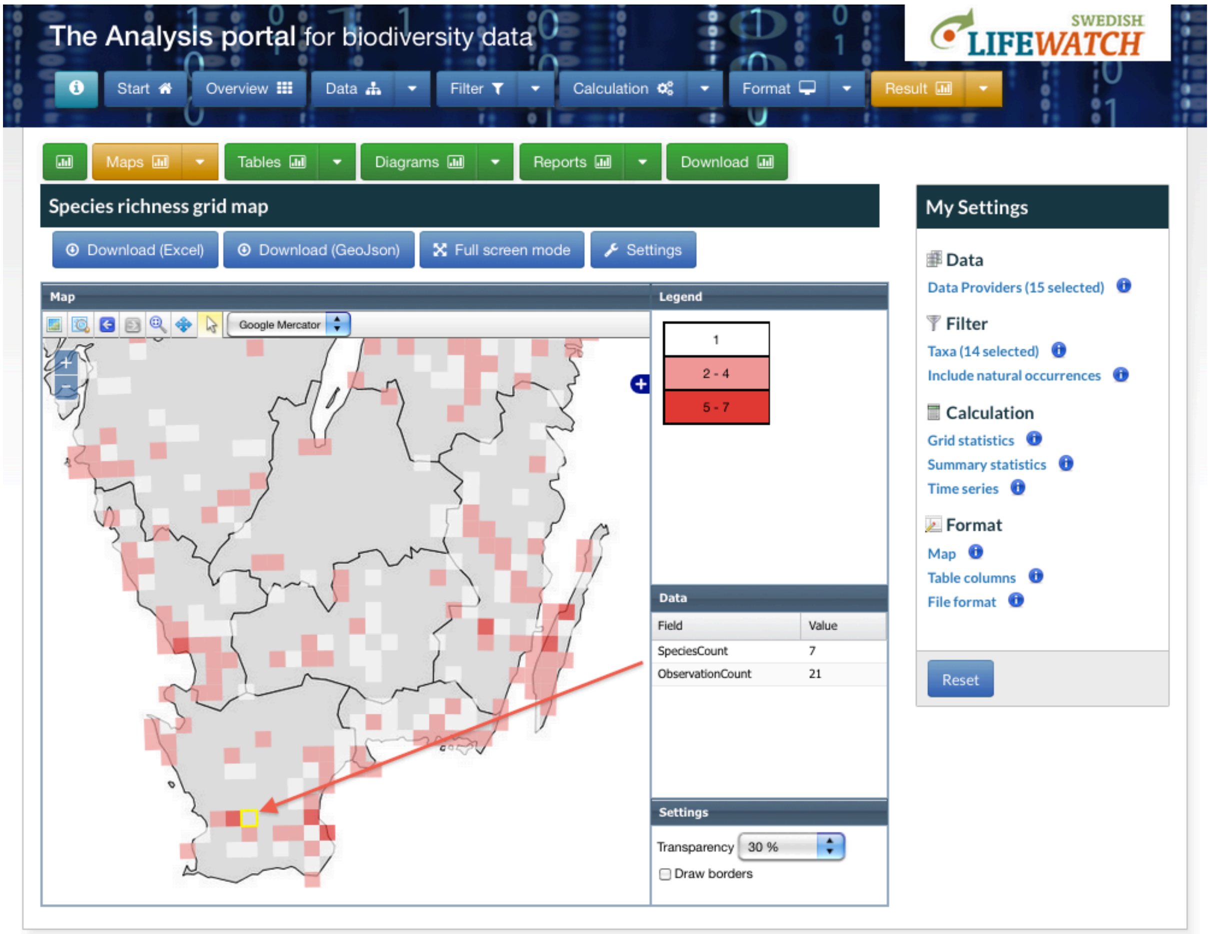

Figure S24: The species richness map for the genus *Lasius*, in the Southern Sweden. The selected grid shows 21 observation records and 7 species counts.

4) As for the species observation data a grid map can also be produced on environmental data. Full screen mode can be chosen and the labels can be set as required. A toolbox for grid parameters at the top allows further settings to be made before the calculation is run. The data panel on the right shows the field and value (Fig. S25). The transparency and drawn borders can be chosen below the data box.

This grid map is rendered based on the grid statistics obtained from core web service method no. 25 (Table 1), which takes the url to the specified environmental data layer (including specified filter statements generated in the Environmental data view) as an input parameter.

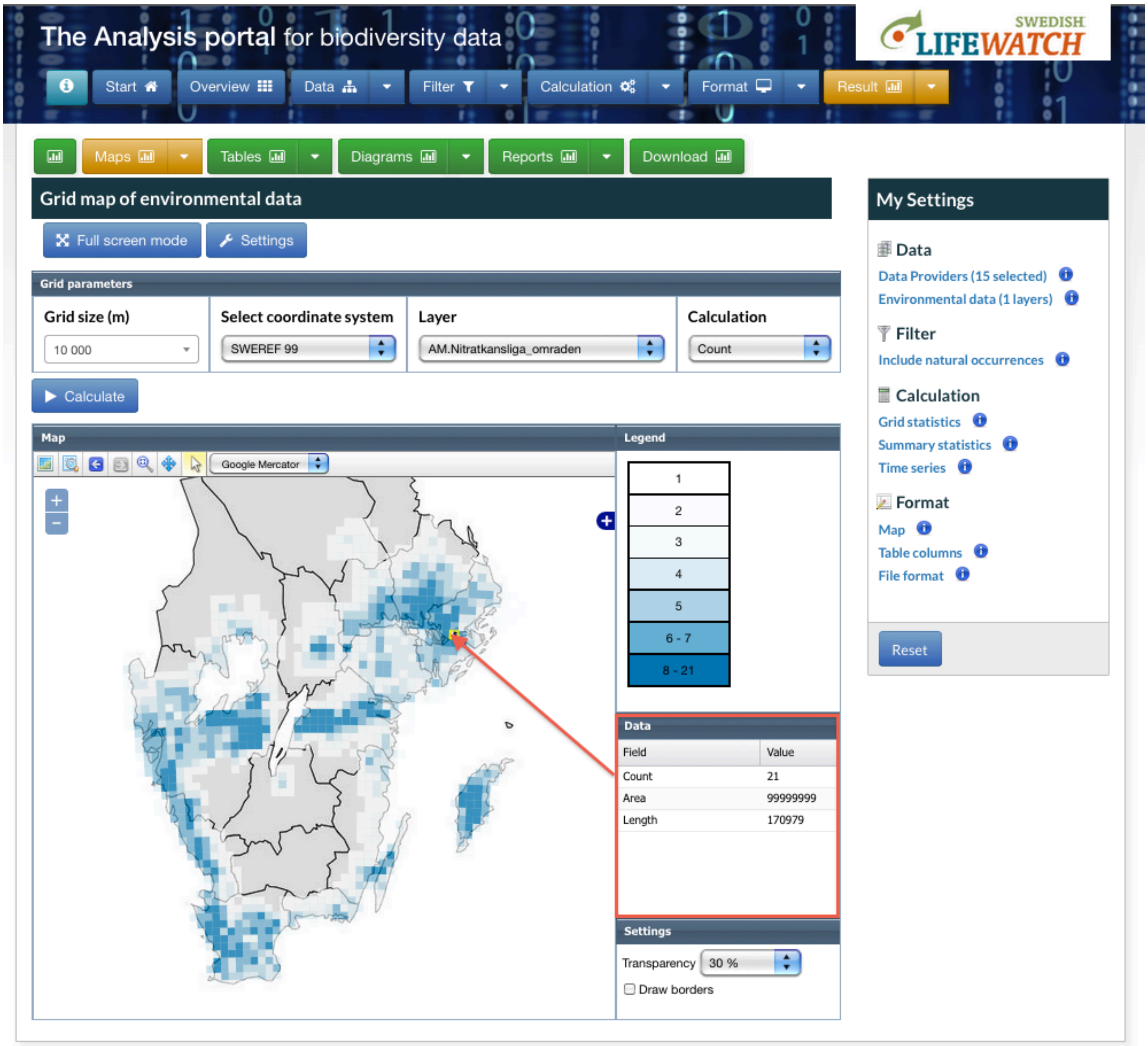

Figure S25: Grid maps on environmental data, here the nitrogen sensitivity in the southern part of Sweden. The Stockholm area shows one of the highest values (21). The layer is uploaded from the Swedish Board of Agriculture.

5) The species observation heat map (> Result – Maps – Species Observation Heat Map) is an image file (.png) that presents the species observations or species richness for the whole of Sweden. The colour scale goes from black (=1) to red (here: >50) (Fig. S26).

This map representation is generated based on the same core web service method that is used for the Species observation grid map, i.e. no. 22 (Table 1).

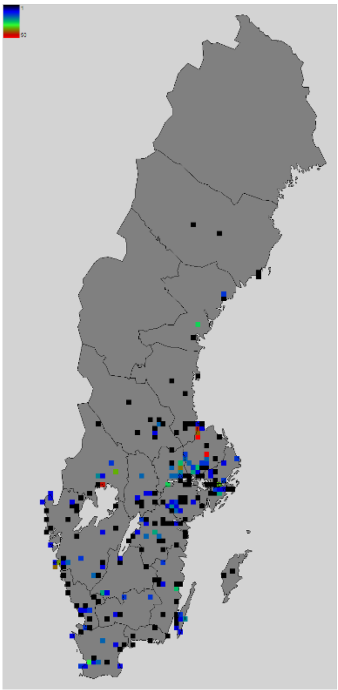

Figure S26: The species observation heat map as image file, here showing the results of the ant *Lasius niger* (colours black = 1 observation; red > 50 observations).

Tables

The AP offers the user seven different types of data table to display and download the results (> Result – Tables).

1) The species observation table presents all the records that fit the specified filter settings of species observations (> Result – Tables – Species observation table). The number and content of the table columns as well as the downloaded file format depend on the format settings that were chosen earlier (see also > Format – Table columns and > Format - File format, respectively) (Fig. S27).

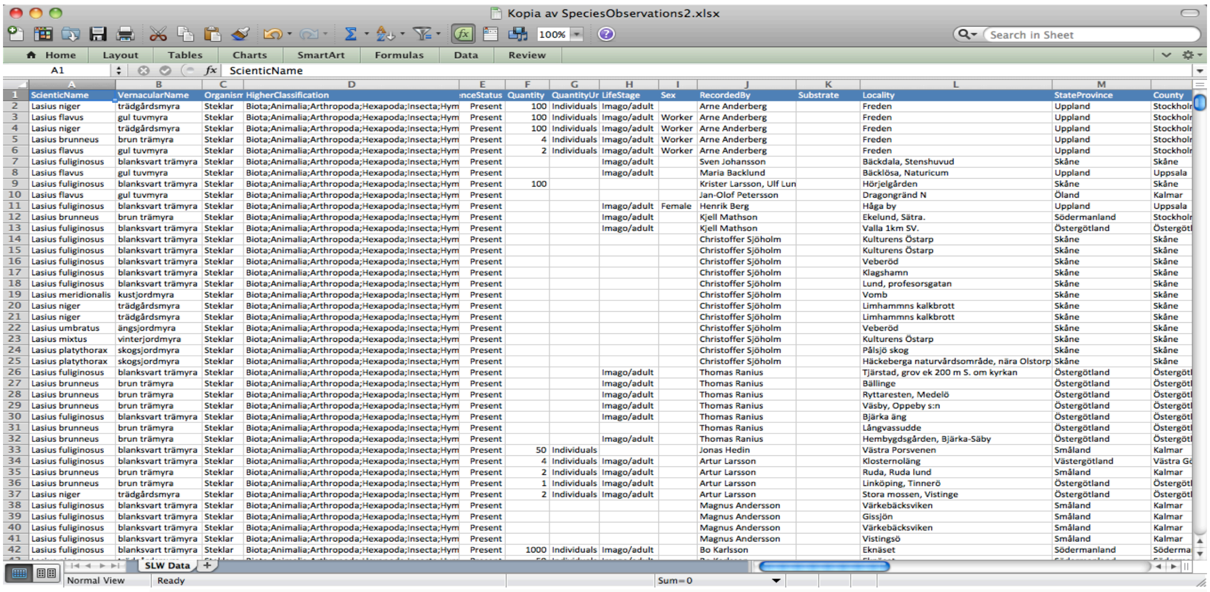

Figure S27: Species observation table for the ant genus *Lasius*. Here, the chosen table format ´basic column set´ and ´Darwin Core labels´.

The observation table view utilizes the same core web service functions that are used behind the Species observation map view, i.e. no. 16 for the table and no. 14 for the detailed representation (Table 1).

2) The observed taxon table lists the taxon, author common name, category and taxon id (> Result – Tables – Observed Taxa). For each column it is possible to sort the results in ascending or descending order (Fig. S28).

This table view is based on core web service function no. 19 (Table 1).

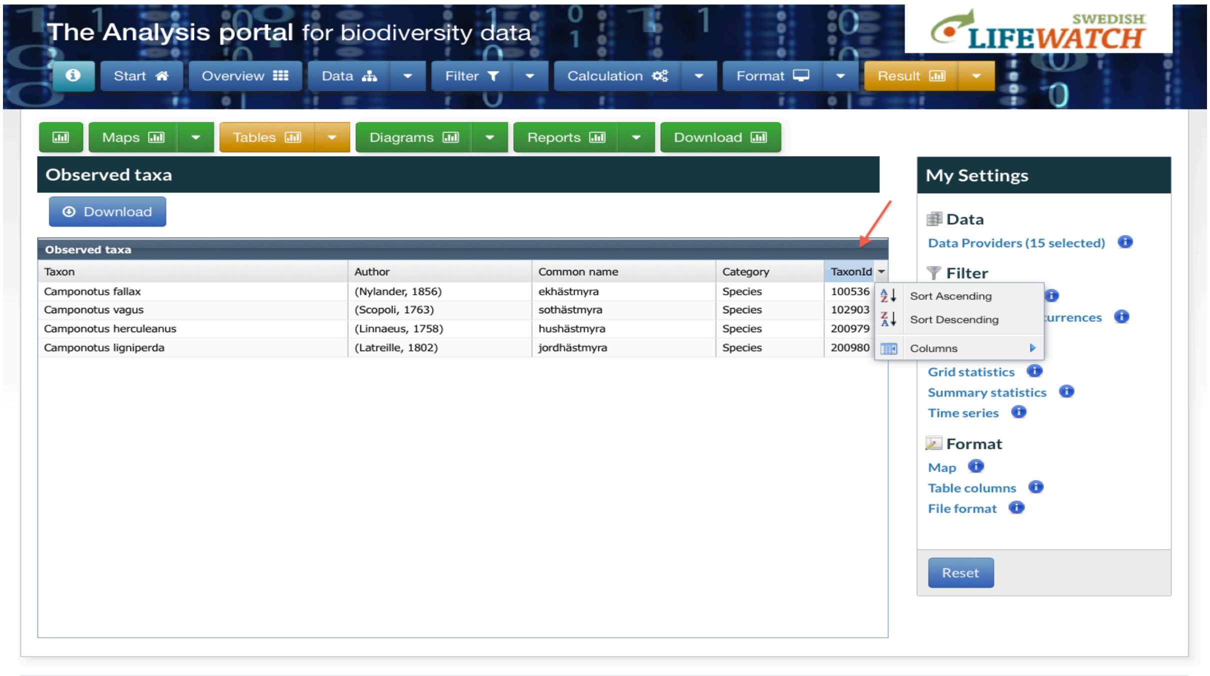

Figure S28: The observed taxa table for the genus *Camponotus* and the possibility to sort data based on a selected column.

3) Another table type is the species observation taxon table with number of observed species (> Result – Tables – Species Observation Taxon With Number of Observed Species Table). In addition to the taxa, the number of observations is given and can be sorted (Fig. S29).

This table view is based on core web service function no. 20 (Table 1).

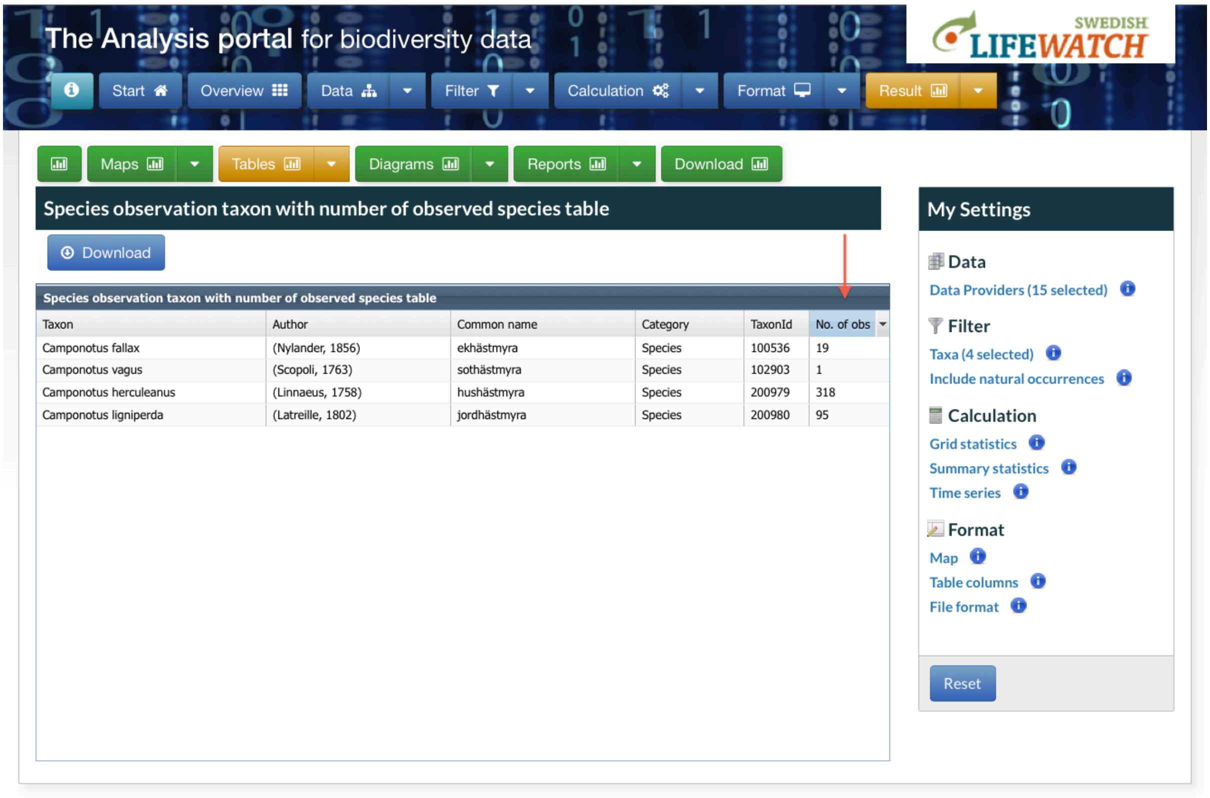

Figure S29: Table on numbers of observation per taxa for the genus *Camponotus* in Sweden. The number of observations is sorted.

4) The grid statistics table on species observation counts (> Result – Tables – Grid Map Statistics Table On Species Observations) gives the id, the observation count and the coordinates in the SWEREF 99 (EPSG:3006) and Google Web Mercator (EPSG:3857) coordinate systems as well as the grid cell width in metres (Fig. S30). Each column can be sorted. The table can be downloaded as an Excel or GeoJSON file.

This table view lists the results obtained from the core web services using function no. 22 (Table 1).

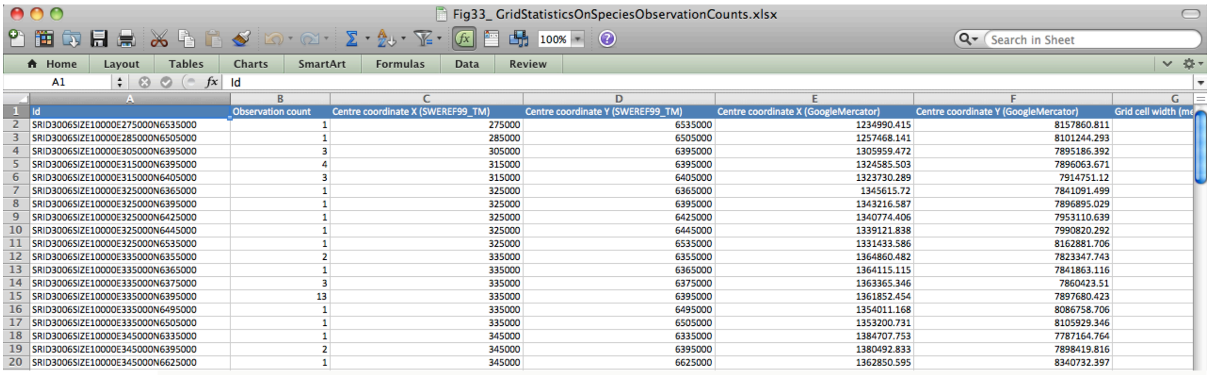

Figure S30: The first 20 rows of the Excel table of grid map statistics on species observations for the ant genus *Camponotus*.

5) In addition to the observation count the grid map statistics on number of species (> Result – Tables – Grid Statistics On Number of Species) also give the species count. Otherwise the table is similar to the grid statistics table on species observation counts, with the same column functions. The data can be downloaded as an Excel and a GeoJSON file (Fig. S31).

This table view lists the results obtained from the core web services using function no. 23 (Table 1).

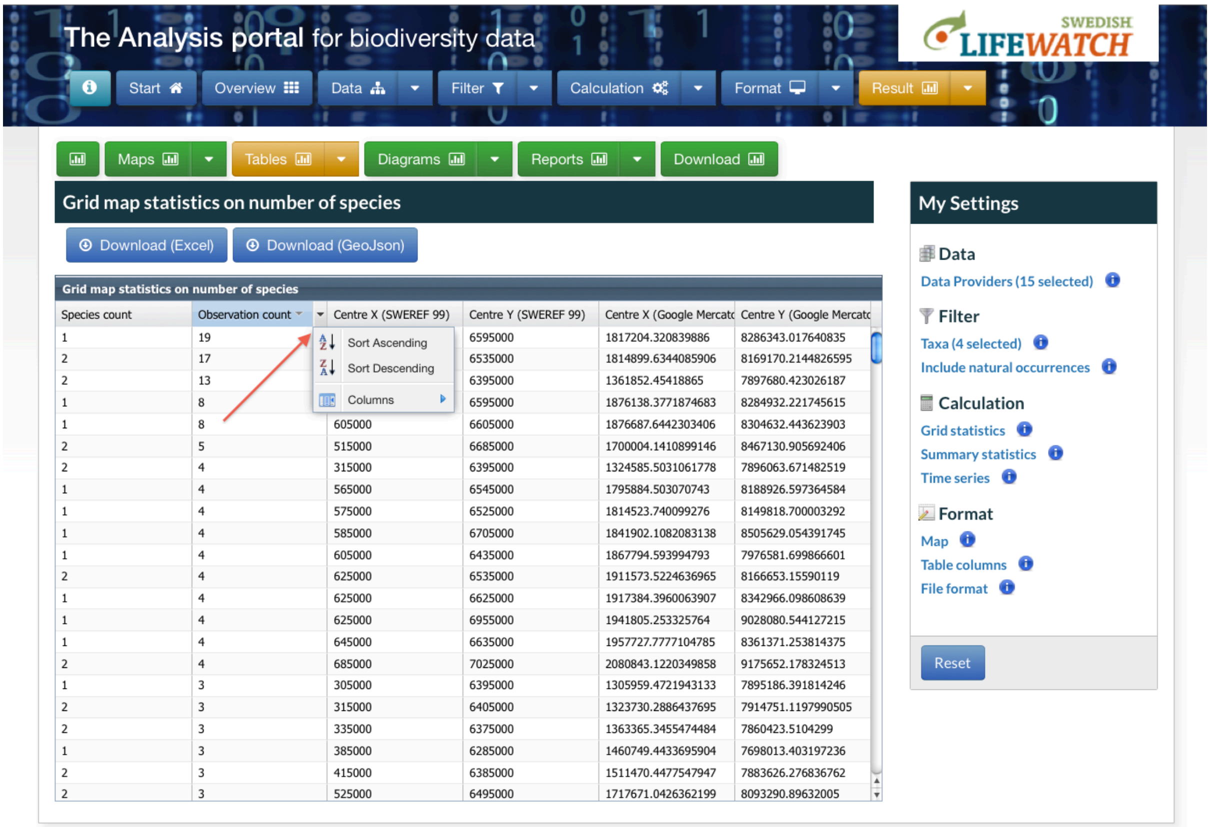

Figure S31: Table on the grid map statistics on number of species, here for the ant species *Camponotus*. It is also shown how to sort the observation counts.

6) The time series table (> Result – Tables – Time Series Table On Species Observation Counts) gives the periodicity (month/week/day of the year, daily, weekly, monthly or yearly)

on species observation counts for the number of taxa selected (Fig. S32). The file can be downloaded as an Excel file.

This table view lists the results obtained from the core web services using function no. 21 (Table 1).

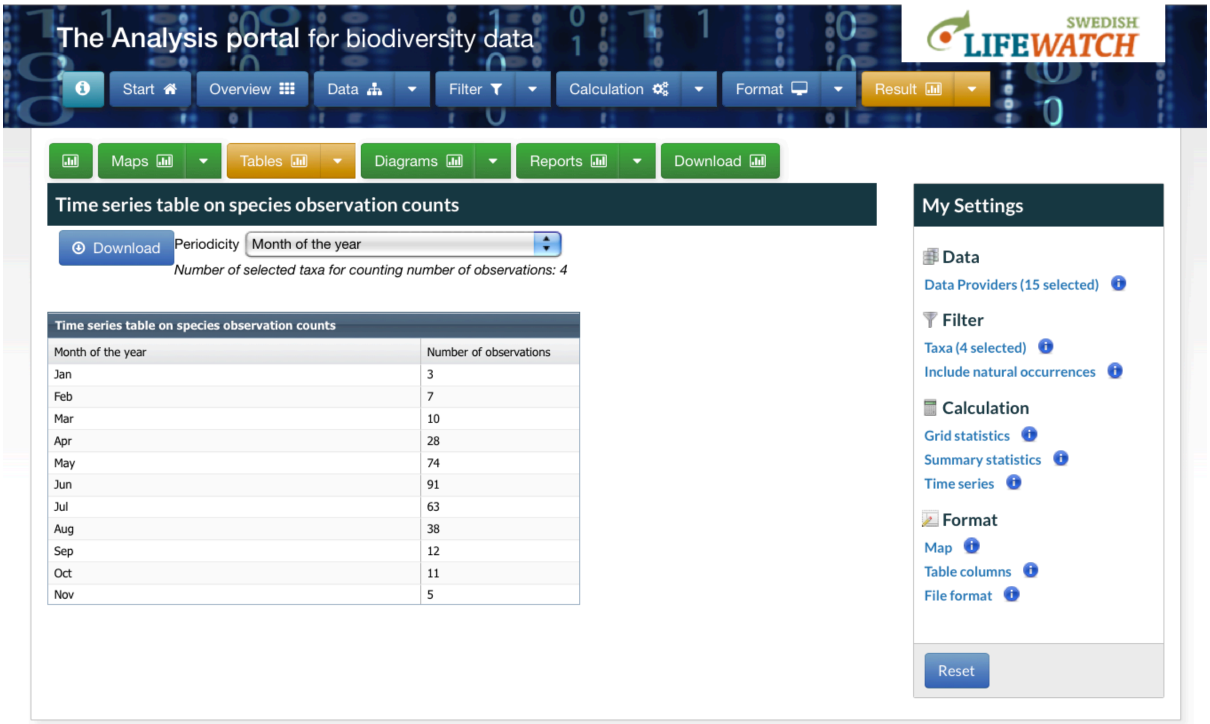

Figure S32: Time series table on species observation counts for the genus *Camponotus*.

7) The summary statistics for the observation and species count per polygon (> Result – Tables – Summary Statistics Per Polygon) can be downloaded as an Excel file if an environmental layer (WFS) is selected beforehand (see also > Calculation – Summary Statistics) (Fig. S33).

The results presented in this table view are obtained by looping through all the features in the specified environmental data layer and repeatedly using core web service function no. 18 (Table 1), where the polygon search criterion (no. 16, Table 2), is set to the current feature’s polygon. The information about the features and their attributes, which are printed in the table, is obtained directly from the WFS.

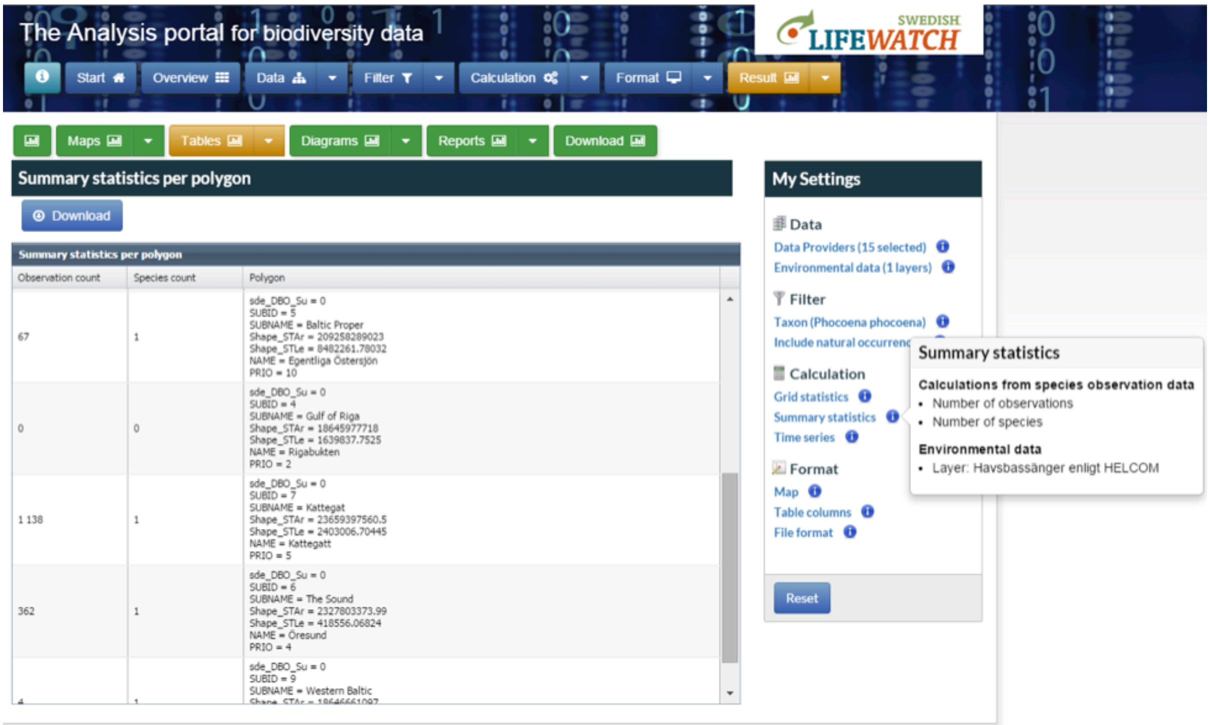

Figure S33: The AP calculates the summary statistics per polygon when a taxon and environmental filter are chosen together. Here is an example for the harbour porpoises (*Phocoena phocoena*) and different sea areas referred to by HELCOM (Baltic Marine Protection Comission - Helsinki Comission).

Diagrams

Two types of diagram are available in the AP.

1) The time series on number of species observations (> Result – Diagrams – Time Series Histogram on Numbers of Observations), where the number of observations will be plotted against their periodicity, which can be chosen between yearly, monthly, weekly, daily and day/week/month of the year (Fig. S34).

The time series is generated based on the results obtained from the core web services using function no. 21 (Table 1).

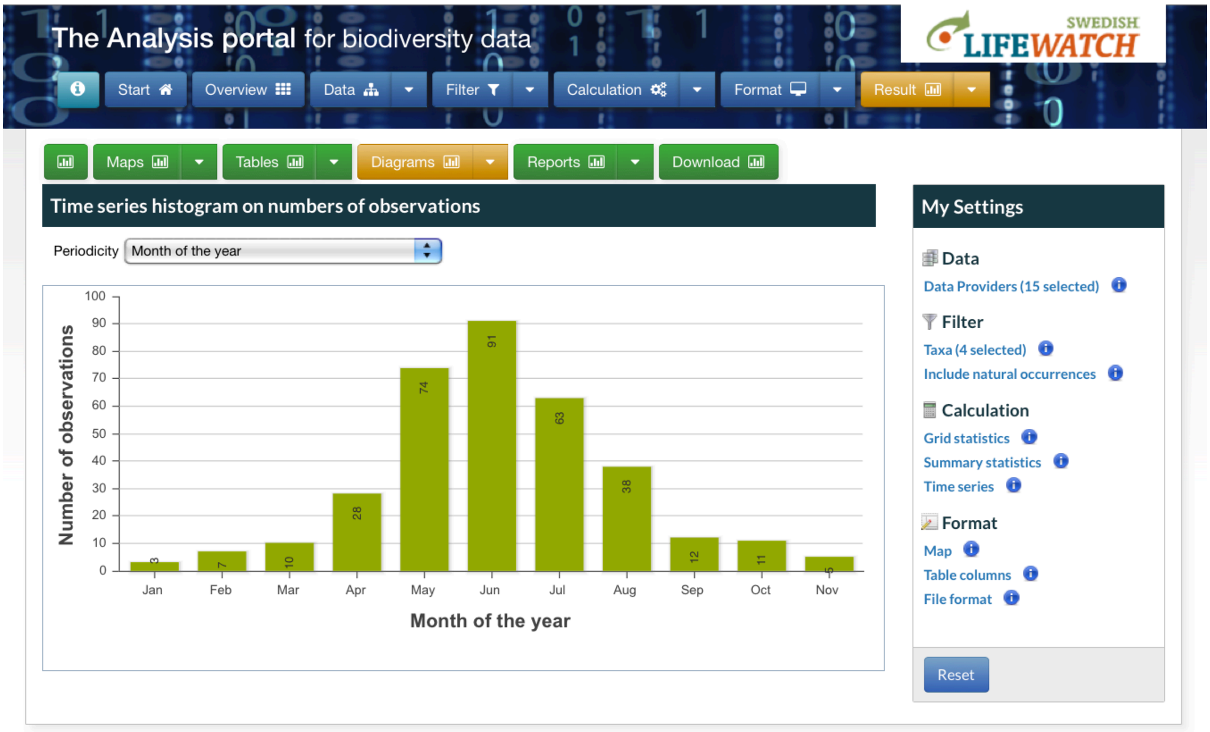

Figure S34: Time series for the four ant species of *Camponotus*. The number of observations is plotted against the month of the year.

2) The time series diagram on taxon-specific abundance index (> Result – Diagram – Time Series Diagram on Taxon Specific Abundance Index) shows the abundance index plotted against a certain periodicity that can be chosen.

When analysing time series of species observation counts in order to try to evaluate population trends of specific species it is most often necessary to adjust estimates for variation in sampling intensity. Of course, if the analysis is restricted to a dataset where the sampling effort has deliberately been kept constant, counts can be interpreted without adjustments. When mixing data from many data providers and data sets, as is the normal case when retrieving data from the AP, it is important to be aware of the fact that sampling intensity varies significantly in both time and space. The general trend is that sampling effort increases over time as the number of observers and the willingness to report increase over time. The rate of increase, however, is not constant and the patterns differ regionally.

The tendency to report species observations that have been made also changes over time and the patterns differ among species and organism groups. For example, bird watchers tend to report their earliest observations of species each year during spring to a greater extent than later observations (Isaac & Pocock 2015, Hurlbert & Liang 2012). Many researches have started to explore different ways to use data from mixed sources for trend analysis. One of the earliest suggested approaches was to calculate an abundance index which adjusts the actual observation count of the focal species to all counts made during the same time periods in the same area of all other species assumed to be reported by the same group of people. If the tendency to report an observation of a species in relation to all other species in the reference group is kept fairly constant over the investigated time period, then the index would be expected to correlate with the actual population trend to a much greater extent than if data are used without any corrections. This correlation is

expected to be stronger when the number of reports increases, the variation in behaviour among reporters decreases, and the detectability of the species increases.

The species and time step (t) specific abundance index, A_t, is calculated based on Eq. 1.

$A_t = \log((S_t/(S_t+T_t))/(S/S+T))$,

where S_t and S are the time step specific and the total count of reported observations of the focal species respectively, while T_t and T are the corresponding counts of reports of all species in the reference group. If the index is zero then the number of observations of the focal species is no different from what is expected on average compared to all species in the reference group. When the index has negative values then the focal species has been reported less than expected. When the index is greater than zero then the focal species has been reported more than expected.

Many scientists are sceptical about the use of this kind of index, arguing that the patterns can be explained by far too many factors than are possible to control for. It is important to use the index with great caution. Nevertheless, by plotting the index for different species in combination with alternative search criteria and reference groups one can find patterns that may generate interesting hypotheses that can be explored further.

The periodicity and selected taxa can be changed through the toolbars at the headline (Fig. S35). It is also possible to download an Excel file. This file contains the taxon id, taxon name, time step, abundance index, count and total count.

Among the core web services there is no function where the abundance index (Eqn. 1) can be retrieved directly. The AP instead uses the same function, which is used to generate the time series plot showing time step specific observation counts, i.e. no. 21 (Table 1). The procedure starts by setting all the selected taxa to the species observation search criteria using the criteria named TaxonIds (no. 1, Table 2) and thereby retrieving all the time step specific total counts (T_t) and the total count (T) of observations. To enable the calculation of the index, a new call to the web service function is required for each species, where the TaxonIds property of the species observation search criteria are set to that particular taxon id. This gives the necessary species specific counts (S_t and S) used in Eqn. 1.

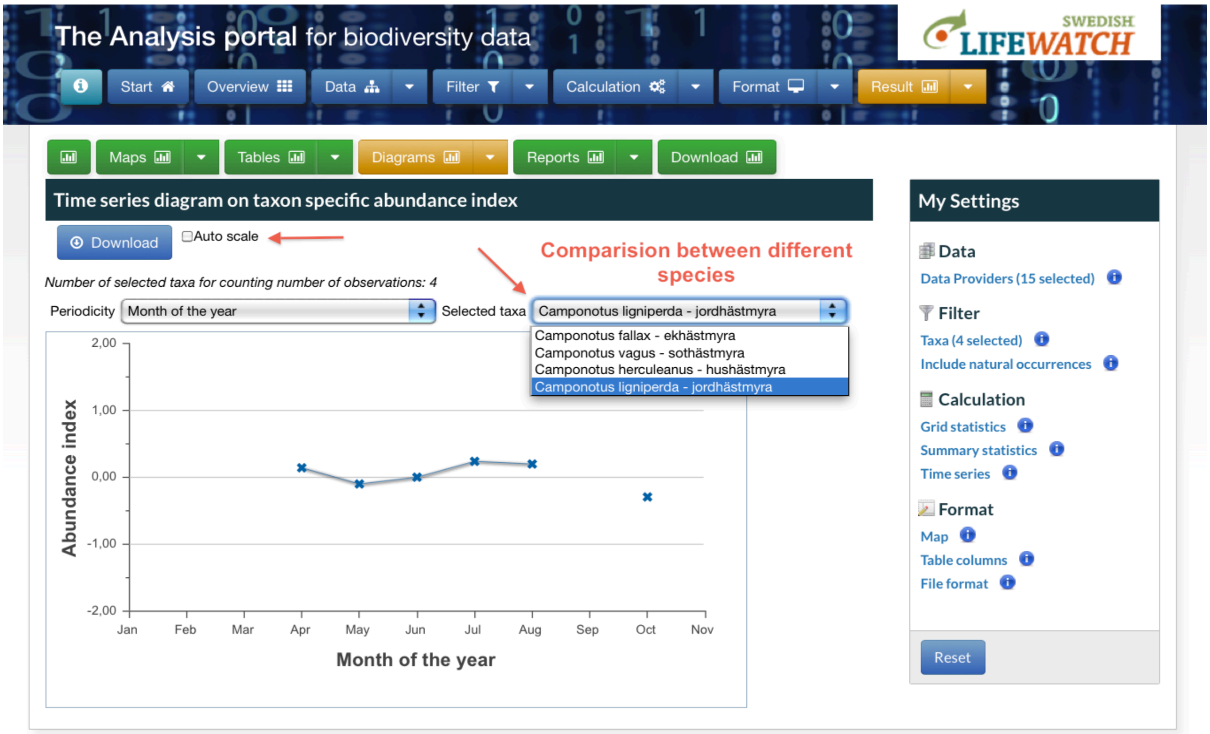

Figure S35: Time series diagram of the taxon-specific abundance index for the ant species *Camponotus ligniperda*. It is possible to adjust the scale automatically.

Reports

The user can download three different kinds of report on the analysis run.

1) The summary statistics report (> Result – Reports – Summary Statistics) is a simple overview of the species observation data the calculations were done on. The report gives the number of observations and number of species only.

The summary statistics presented in this report view are obtained from the core web services using function no. 18 (Table 1), which delivers both the number of observations and the number of observed species.

2) The species observation provenance report (> Result – Reports – Species Observation Provenance) shows the owner, observer, reporter and data provider for all species observations that were used in the analysis (Fig. S36).

This report is generated based on the results obtained from core web services function no. 24 (Table 1).

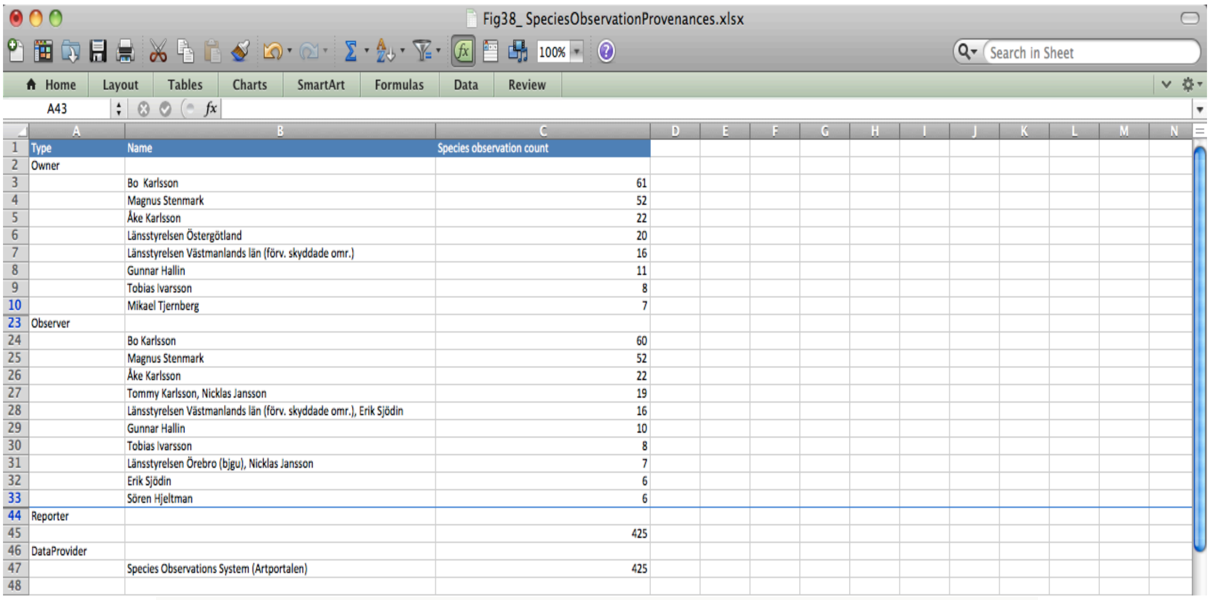

Type	Name	Species observation count
Owner		
	Bo Karlsson	61
	Magnus Stenmark	52
	Åke Karlsson	22
	Länsstyrelsen Östergötland	20
	Länsstyrelsen Västmanlands län (förv. skyddade omr.)	16
	Gunnar Hallin	11
	Tobias Ivarsson	8
	Mikael Tjernberg	7
Observer		
	Bo Karlsson	60
	Magnus Stenmark	52
	Åke Karlsson	22
	Tommy Karlsson, Nicklas Jansson	19
	Länsstyrelsen Västmanlands län (förv. skyddade omr.), Erik Sjödin	16
	Gunnar Hallin	10
	Tobias Ivarsson	8
	Länsstyrelsen Örebro (bjgu), Nicklas Jansson	7
	Erik Sjödin	6
	Sören Hjeltman	6
Reporter		
		425
DataProvider		
	Species Observations System (Artportalen)	425

Figure S36: The species observation provenance report for the ant genus *Camponotus*.

3) The setting report (> Result – Reports – Setting Report) gives an overview of the selected settings during the analysis. The tables give information about the selected data providers, the chosen filter(s), the grid statistics, summary statistics and time series settings (Fig. S37).

All three reports can be downloaded as Excel files.

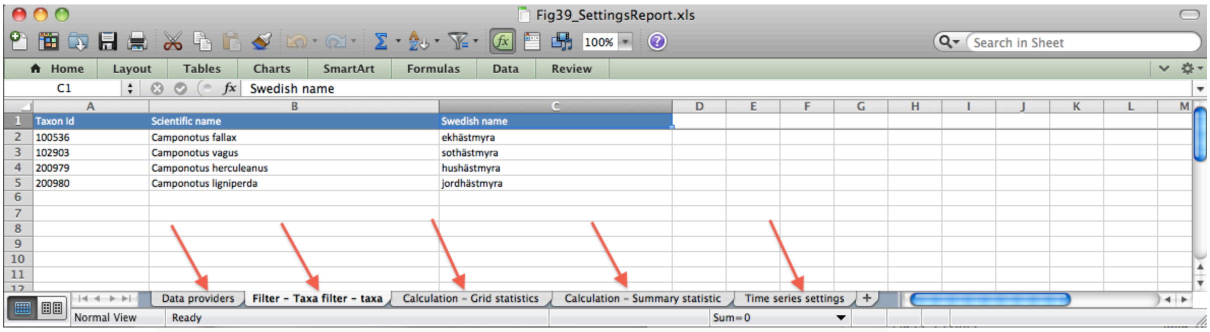

Taxon Id	Scientific name	Swedish name
100536	Camponotus fallax	ekhästmyra
102903	Camponotus vagus	sothästmyra
200979	Camponotus herculeanus	hushästmyra
200980	Camponotus ligniperda	jordhästmyra

Figure S37: The setting report as excel file for the ant genus *Camponotus*.

Downloads

At present, there are numerous options to download data sets and analysis results from the AP. The five main groups are Excel tables, comma-separated values, images/graphics and other formats (Fig. S38).

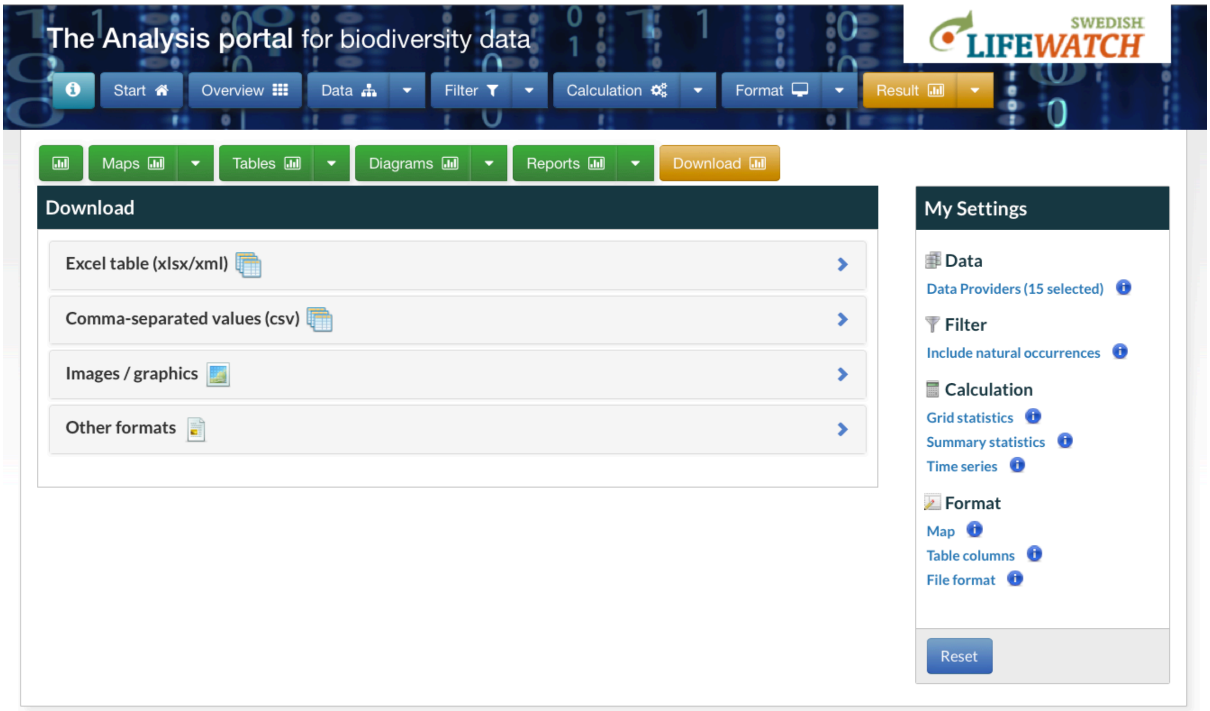

Figure S38: There are several download functions (Excel tables, comma-separated values, image/graphics, other format) as well as workflow functions.

A) Excel tables are available in .xlsx or .xml format. At present, the user has the possibility to download 16 different types of table (> Result - Downloads – Excel Table), which are listed below:

1) Data providers (see also > Result - Reports - Setting Report)

2) Species observations (see also > Result – Tables – Species Observation Table)

3) Observed taxa (see also > Result – Tables – Observed Taxa)

4) Number of observations per taxon (see also > Result – Tables – Species Observation Taxon With Number of Observed Species Table)

5) Grid map statistics on species observations (see also > Result – Tables – Grid Statistics Table On Species Observations)

6) Grid map statistics on number of species (see also (> Result – Tables – Grid Statistics On Number of Species)

7) Taxon specific grid observation counts

This download method does not correspond to any of the graphical visualizations. It is designed to support calculations of different kinds of biodiversity index. Based on the species observation search criteria, current grid calculation settings and the list of taxa

specified with the taxon filter, this function creates a two-dimensional matrix with the taxa listed as columns and the grid cells as rows. The columns are labelled by the ScientificName and the DyntaxaTaxonID and the rows are labelled with a grid cell identifier which is constructed based on SRID (Coordinate system id), grid cell size in metres and the eastern and western coordinates of the grid cell central point. For each combination of grid cell and taxon the matrix holds a value that represents the number of observed records.

The calculations behind this download are done by the core web services using the same method (no. 22, Table 1) as that used behind the Species observation grid map. For each taxon in the list a new call to the web service is made that sets the TaxonIds property (no. 1, Table 2) of the search criteria to the taxon id of the taxon.

8) Taxon specific grid occurrence

This download method is practically identical to download method A7. However, in this case the matrix holds a value that represents the observed occupancy for each combination of grid cell and taxon.

The calculations behind this download are done by the core web services using the same method (no. 22, Table 1) as that used behind the species observation grid map. For each taxon in the list a new call to the web service is made that sets the TaxonIds property (no. 1, Table 2) of the search criteria to the taxon id of the taxon. If the grid cell and taxon-specific count of observations is greater than 0, the occupancy is set to 1. Otherwise the occupancy is set to 0.

9) Time series table on species observation counts (see also > Result – Diagrams – Time Series Histogram on Numbers of Observations)

10) Time series on taxon abundance index (see also > Result – Diagram – Time Series Diagram on Taxon Specific Abundance Index)

11) Combined grid statistics from species observations and environmental data

This download method does not correspond to any of the graphical visualizations. However, it combines the output of download nos. 6 and 11.

12) Summary statistics per polygon (see also > Result – Tables – Summary Statistics Per Polygon)

13) Taxon specific occurrence per polygon

This method is similar to download function no. A8, but instead of grid cells it loops through the polygons in the filtered WFS data layer estimate occupancy based on the retrieved observation counts. To retrieve the species observation counts per polygon, which the occupancy values are based upon, core web service method no. 17 (Table 1) is used repeatedly for each combination of taxon and polygon.

14) Taxon specific species observation count per polygon

This method is similar to download function no. A7, but instead of grid cells it loops through the polygons in the filtered WFS data layer. To retrieve the species observation counts per polygon core web service method no. 17 (Table 1) is used repeatedly for each combination of taxon and polygon.

15) Summary statistics (see also > Result – Reports – Summary Statistics)

16) Species observation provenance (see also > Result – Reports – Species Observation Provenance)

B) Comma-separated values (csv) (> Result - Downloads – Comma-separated values)

1) Species observation table (see also > Result – Tables – Species Observation Table)

2) Taxon specific grid observations counts (see Download method A7).

3) Taxon specific grid occurrence (see Download method A8).

C) Images/graphics (png) (> Result - Downloads – Images/Graphics)

1) Species observation grid map (see also > Result – Maps – Species Observation Map)

2) Species richness grid map (see also > Result – Maps – Species Richness Grid Map)

D) Other formats (GeoJSON) (> Result - Downloads – Other formats)

1) Grid map statistics on species observations (see also > Result – Tables – Grid Statistics Table On Species Observations)

References:

Hurlbert A, Liang Z (2012) Spatiotemporal Variation in Avian Migration Phenology: Citizen Science Revals Effects of Climate Change. PLoS ONE 7 (2): e31662. DOI: 10.1371/journal.pone.0031662

Isaac NB, Pocock MO (2015) Bias and information in biological records. Biological Journal of the Linnean Society 115(3): 522-531. DOI: 10.1111/bij.12532

IUCN (2001) IUCN Red List Categories and Criteria: Version 3.1. IUCN Species Survival Commission, 3.1. IUCN, Gland, Switzerland and Cambridge, UK:, 30 pp. URL: http://www.iucnredlist.org/technical-documents/categories-and-criteria/2001-categories-criteria
